# Independent evolution of satellite DNA sequences in homologous sex chromosomes of Neotropical armored catfish (*Harttia*)

**DOI:** 10.1038/s42003-025-07891-6

**Published:** 2025-03-30

**Authors:** Francisco de M. C. Sassi, Manuel A. Garrido-Ramos, Ricardo Utsunomia, Rodrigo Zeni dos Santos, Tariq Ezaz, Geize A. Deon, Fábio Porto-Foresti, Thomas Liehr, Marcelo de B. Cioffi

**Affiliations:** 1 Laboratory of Evolutionary Cytogenetics, Department of Genetics and Evolution, Universidade Federal de São Carlos, São Carlos, SP 13565-905 Brazil; 2 Departamento de Genética, Facultad de Ciencias, Universidad de Granada, 18071 Granada, Spain; 3 Faculdade de Ciências, UNESP, Bauru, SP 17033-360 Brazil; 4 Institute for Applied Ecology, University of Canberra, Canberra, ACT 2617 Australia; 5 Universitätsklinikum Jena, Friedrich-Schiller Universität, Institut für Humangenetik, Jena, Thüringen 07747 Germany

**Keywords:** Cytogenetics, Evolutionary genetics

## Abstract

The Neotropical armored catfish *Harttia* is a valuable model for studying sex chromosome evolution, featuring two independently evolved male-heterogametic systems. This study examined satellitomes—sets of satellite DNAs—from four Amazonian species: *H. duriventris* (X_1_X_2_Y), *H. rondoni* (XY), *H. punctata* (X_1_X_2_Y), and *H. villasboas* (X_1_X_2_Y). These species share homologous sex chromosomes, with their satellitomes showing a high number of homologous satellite DNAs (satDNAs), primarily located on centromeres or telomeres, and varying by species. Each species revealed a distinct satDNA profile, with independent amplification and homogenization events occurring, suggesting an important role of these repetitive sequences in sex chromosome differentiation in a short evolutionary time, especially in recently originated sex chromosomes. Whole chromosome painting and bioinformatics revealed that in *Harttia* species without heteromorphic sex chromosomes, a specific satDNA (HviSat08-4011) is amplified in the same linkage group associated with sex chromosomes, suggesting an ancestral system. Such sequence (HviSat08-4011) has partial homology with the *ZP4* gene responsible for the formation of the egg envelope, in which its role is discussed. This study indicates that these homologous sex chromosomes have diverged rapidly, recently, and independently in their satDNA content, with transposable elements playing a minor role when compared their roles on autosomal chromosome evolution.

## Abstract (web summary)

The study on Neotropical armored catfish Harttia explores sex chromosome evolution in four Amazonian species with male-heterogametic systems, revealing that homologous sex chromosomes have species-specific satellite DNA variations.

## Introduction

Multiple sex chromosome systems arise from rearrangements between XY or ZW chromosomes and autosomes, this being the most common way, observed in several animal and plant groups^1–5^, or even from fissions within the ancestral sex pairs without the involvement of additional autosomes^6–12^. In contrast, the differentiation of sex chromosomes in simple sex chromosome systems (i.e., XX female/XY male; ZZ male/ZW female), which is based on autosomal ancestry, is more extensively clarified^13–17^. An autosomal pair generally acquires a sex-determining locus, which may be a dosage-dependent or sex-determining allele. Subsequent modifications include the suppression of recombination either via the accumulation of various repetitive DNA classes or via chromosome rearrangements^18–23^. Undoubtedly, compared to the extensive research that has already been done on simple XY and ZW systems, multiple systems still have significant shortcomings in evolutionary research.

While some taxa, like birds and mammals, have retained the same mode of sex chromosome system (i.e., ZW and XY, respectively) in most of their extant species throughout their evolutionary history, fish and amphibians show regular turnovers, with a large number of species presenting homomorphic, simple and multiple sex chromosomes, including alternative modes of sex chromosome system even within closely related species^5,24–27^. Among vertebrates, multiple sex chromosome systems derived from the ZZ/ZW system are extremely rare, only known in a few birds, lizards, and fish species (revised in ref. ^5^). On the other hand, the X_1_X_1_X_2_X_2_/X_1_X_2_Y system is the most widespread XY-derived system, which has been found in several mammals and reptiles, frogs, and fishes^3,5^. Fishes possess the most abundant and diverse range of multiple sex chromosome systems among vertebrates. Approximately 5% of the teleost species examined had heteromorphic sex chromosomes, with a total of 81 instances of multiple systems^2,4,5,28–31^.

**Fig. 1:**
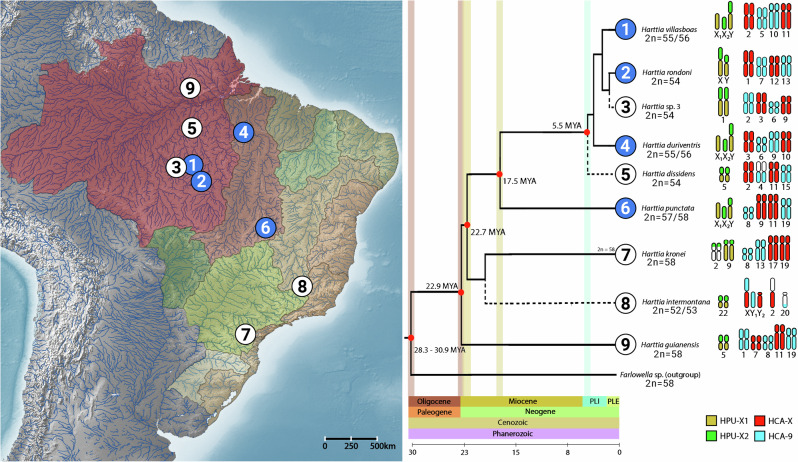
Distribution map of *Harttia* species herein investigated, with their respective phylogenetic relationships modified from Covain et al.^70^. The map highlights the hydrographic basins of Brazil and the rivers of South America. Dashed lines indicate the inferred relationships of species based on chromosomal data^10,11,34,87,90,124^ that are not included in the previous^70^ phylogeny. Numbers on the map indicate the collection sites (detailed in the material and methods section Supplementary Table 11) of the same species presented in phylogeny. Idiograms illustrate the homologous sex chromosome systems according to their linkage groups, following previous whole-chromosome painting experiments^10,11,34,35^. Nodes marked in red indicate the divergence between clades recovered from TimeTree 5^141^. Blue dots indicate the species targeted for satellitome characterization, while the white dots indicate the ones used for comparative FISH experiments.

*Harttia* (Siluriformes, Loricariidae) is a Neotropical fish genus with significant karyotype diversity among its 28 recognized and several cryptic species, which are widespread in the waters of the Orinoco and Guyana shields, as well as most Brazilian rivers^32,33^. The genus *Harttia* records the highest number of species with multiple sex chromosomes among fishes, with six male-heterogametic occurrences, comprising two independently evolved sex chromosome systems: XX/XY_1_Y_2_ and X_1_X_1_X_2_X_2_/X_1_X_2_Y (Supplementary Table 1, Fig. 1). Whole chromosome painting (WCP) studies combined with the fluorescence in situ (FISH) mapping of repetitive sequences such as ribosomal DNA (rDNA) and microsatellites highlighted the putative origins and evolutionary relationships of the sex chromosomes, indicating that independent fission events occurred in the origin of the multiple systems^10,11,34,35^.

SatDNAs are fast-evolving repetitive sequences that show species-specific profiles^36,37^. Notwithstanding, these sequences might have a particular role in gene regulation, chromatin modification, or as chromosomal functional components^36,38^. The extensive mapping of repetitive DNA sequences in fish chromosomes has provided an unparalleled opportunity to understand the role of such sequences in the evolutionary process^39^. Although fish genomics has been studied since the late 1980s, the broad incorporation of next-generation sequencing methods in the study of non-model organisms (e.g. refs. ^40–43^), allowed the expansion of chromosomics^44^. More recently, this combination of techniques (cytogenetics and genomics) is being used to characterize and map full catalogs of satellite DNA sequences (satDNAs) in several non-model fish (e.g. refs. ^41,45–48^), revealing the essential role of those repetitive sequences in sex chromosomal differentiation^49–53^.

In standard XX/XY and ZZ/ZW systems, the association of satDNA sequences with the differentiation of the sex-specific (Y or W) chromosome has been clearly demonstrated (e.g. refs. ^49,50,52,54–59^). The few studies on the satDNA content of multiple sex chromosome systems also demonstrate an accumulation of these repetitive sequences in distinct regions of the sex chromosomes, as demonstrated for the cricket *Eneoptera surinamensis* (X_1_X_2_Y) that carries satDNAs in the Y^60^ and the anostomid *Megaleporinus elongatus* (Z_1_W_1_Z_2_W_2_) whose accumulation occurs in the W_1_^41^. The sorrel plant (*Rumex acetosa*, XY_1_Y_2_) presents satDNAs accumulated at both Y_1_ and Y_2_ sex chromosomes with a different satDNA profile from those at the X chromosome, suggesting an older origin when compared to other dioecious plants with simple sex chromosomes^61–67^. The *Oxycarenus hyalinipennis* (X_1_X_2_Y) bug has a satDNA family distributed through the entire length of the Y chromosome and at least 26 satDNA families with a bias towards the male genome^68^. Although lacking Y-specific motifs, several satDNAs of *Pyrrhulina semifasciata* (X_1_X_2_Y) are accumulated in the proto-sex pairs in relative species^69^. Except for the few studies described above, no research on fish has been reported comparing species satellitomes with homologous multiple sex chromosomes. In the absence of chromosome-scale genome assemblies for the genus (and the whole family), we integrate genomic and chromosomal data to uncovered and compared the complete catalogs of satDNA families of four *Harttia* species named *H. duriventris, H. punctata*, and *H. villasboas* (X_1_X_2_Y) and *H. rondoni* (XY). This combined approach allowed us to investigate the evolutionary trajectory of satDNA families and expose their recent and accelerated patterns of evolution in these rearranged multiple sex chromosomes.

## Results

### Characteristics of Harttia satellitomes

The main features of the satellitomes are compiled in Table 1 and Supplementary Data 1, including the number of satellite sequences, the maximum and minimal values for the A + T proportion, and the range of repeat unit lengths. The maximum number of satellites was recovered in *H. rondoni*, with 25 satDNA families. This species also has the largest variation in the repeat unit length, with satDNAs varying from 4165 bp to 19 bp. Long satDNAs predominate among the four satellitomes, corresponding to 76.2% of the sequences in *H. villasboas*, 64% in *H. rondoni*, 61.2% in *H. duriventris*, and 52.6% in *H. punctata*. Putative ORFs were identified in four HviSatDNAs, five in HroSatDNAs, three in HduSatDNAs, and four in HpuSatDNAs (Supplementary Table 3). We have further investigated the satellitome of *H. villasboas* and studied it comparatively with the rest of the species.

**Table 1:** Main features of the *Harttia* satellitomes herein obtained

Species	*N*	Max RUL	Min RUL	Med RUL	Max A + T	Min A + T	Med A + T
*Harttia duriventris*	18	2707	21	262	81%	23.6%	56%
*Harttia punctata*	19	2750	21	177	72%	39%	59%
*Harttia villasboas*	21	4011	32	730	68.1%	24.2%	52%
*Harttia rondoni*	25	4165	19	372	81%	25.1%	57.8%

*N* number of satellite sequences, *RUL* repeat unit length (bp), *A + T* proportion of A + T nucleotides in the satellite, *Max* maximum, *Min* minimal, *Med* median.

### Comparative fluorescence in situ hybridization

**Fig. 2:**
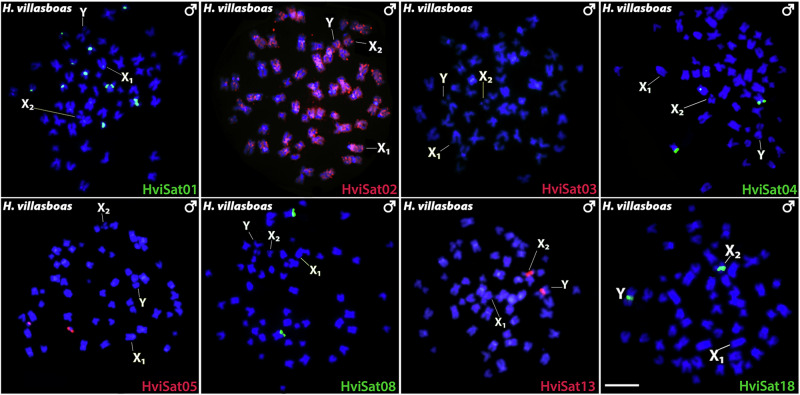
Distribution of satDNAs in *H. villasboas.* Male mitotic chromosomes of *H. villasboas* after hybridization with distinct satellite DNA sequences, indicated in the lower right corner. Sex chromosomes are indicated in the metaphase. Scale bar = 5 µm.

The FISH mapping of satellite sequences revealed distinct patterns for each *Harttia* species. Here we present the results in males, while hybridizations on females are presented in Supplementary Figs. 2 and 3. In the donor species *H. villasboas* (Fig. 2), concerning the sex chromosomes, only HviSat13-730 and HviSat18-1068 were present both at the secondary constriction of X2 and Y. Among autosomes, HviSat01-2531 was found in the pericentromeric regions of five chromosome pairs, while HviSat02-2707 was scattered in all pairs in a non-clustered pattern. Both HviSat04-2959 and HviSat05-177 were hybridized in the short arms of a small metacentric pair, and HviSat08-4011 was found in the long arms at the terminal region of a submetacentric pair. No visible signals were identified with the HviSat03-997 probe.

**Fig. 3:**
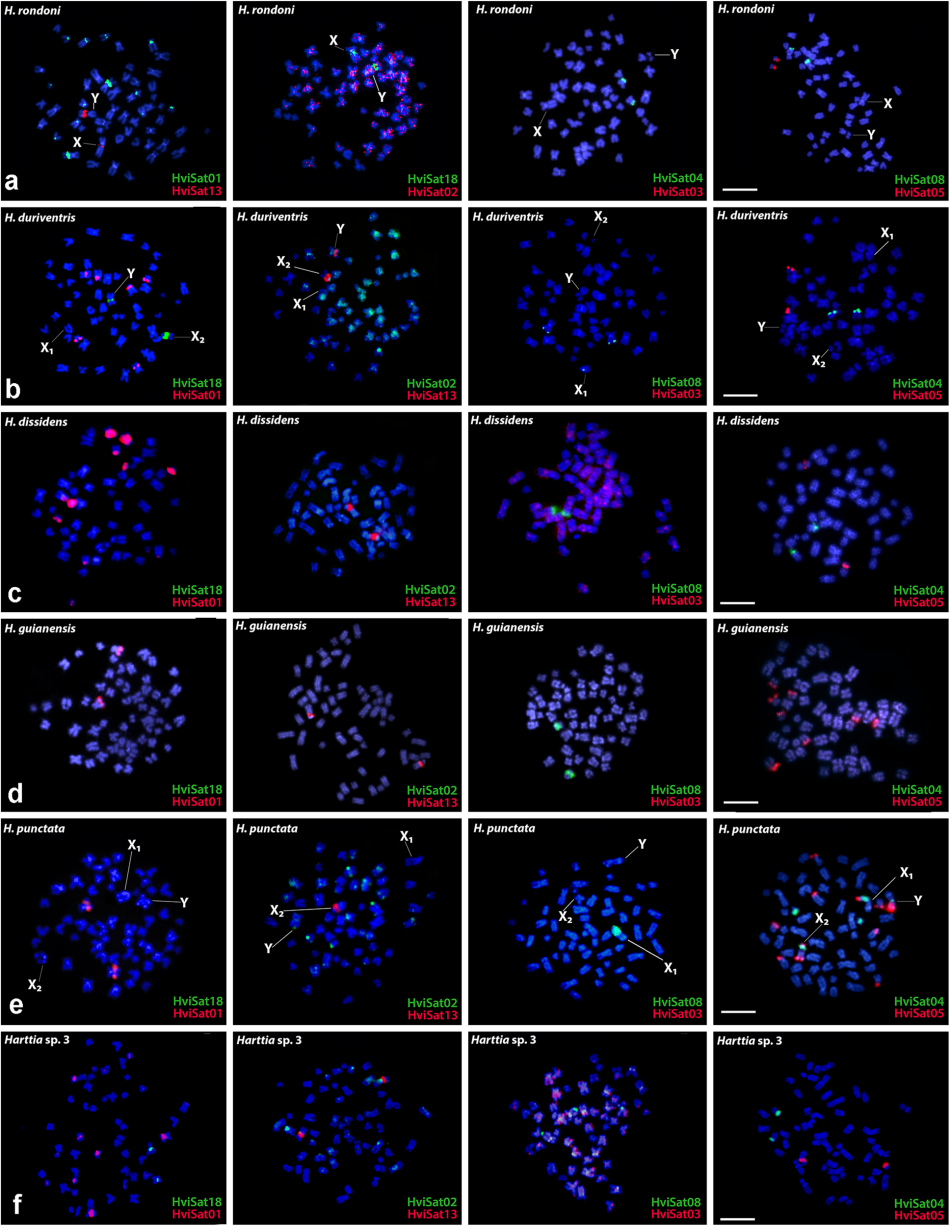
Comparative satDNA mapping in *Harttia.* Male mitotic chromosomes of **a** *H. rondoni*, **b** *H. duriventris*, **c** *H. dissidens*, **d** *H. guianensis*, **e** *H. punctata*, and **f** *Harttia* sp. 3, after hybridization with distinct satellite DNA sequences from *H. villasboas*, as depicted in the lower right corner. Sex chromosomes are indicated. Scale bar = 5 µm.

Both *H. rondoni* and *H. duriventris* share similar distribution patterns of HviSatDNAs. Only HviSat13-730 and HviSat18-1068 were mapped in the X and Y sex chromosomes of *H. rondoni* and X_2_ and Y of *H. duriventris* (Fig. 3a, b). HviSat08-4011 was hybridized to the terminal portion of the long arms of a submetacentric pair, in addition to a pericentromeric signal on the X_1_ chromosome of *H. duriventris*. Following this, HviSat04-2959 and HviSat05-177 were found on the short arms of a small metacentric pair. HviSat02-2707 was found scattered in all chromosomes and HviSat03-997 does not produce visible signals, while HviSat01-2531 was hybridized to pericentromeric regions of six and three pairs in *H. rondoni* and *H. duriventris*, respectively.

**Fig. 4:**
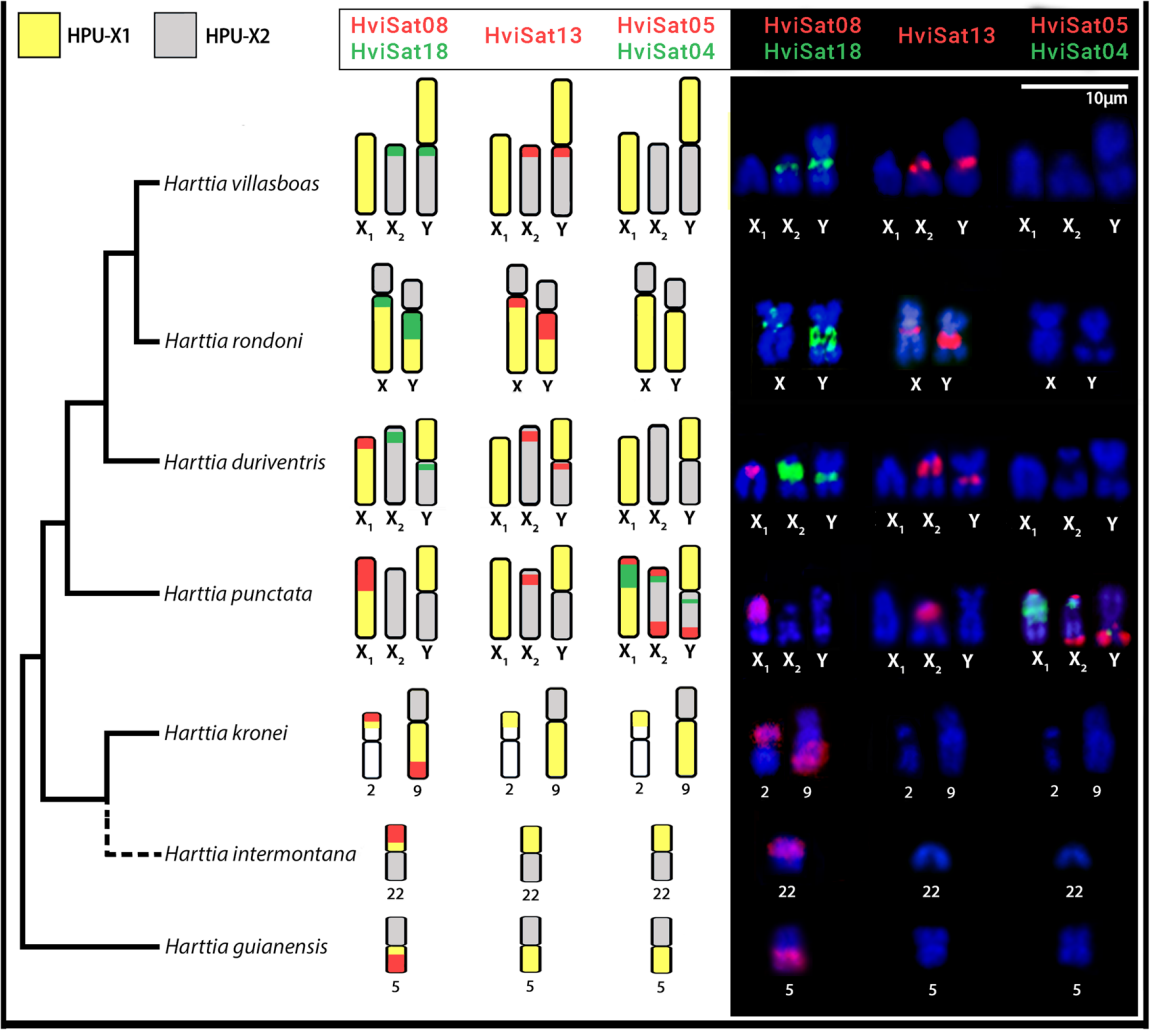
HviSatDNAs hybridized to sex chromosomes and homologous autosomes of seven *Harttia* species. A highlight of *H. villasboas* satellite sequences that were hybridized to sex chromosomes in distinct selected *Harttia* species schematized according to their phylogenetic relationships^70^. *H. intermontana* was manually added to the tree based on geographical distribution and chromosomal characteristics, but its position must be confirmed since it was not included in Covain’s analysis. The combination of probes is indicated above each ideogram/FISH image. The colors of idiograms followed the homology of sex chromosomes obtained from whole chromosome painting with HPU-X_1_ (yellow) and HPU-X_2_ (gray) described by Deon et al.^10,34^ and^11^. See Supplementary Fig. 4 for WCP + HviSat08-4011 on species without heteromorphic sex chromosomes. Scale bar = 10 µm.

For *H. dissidens* and *H. guianensis* (Fig. 3c, d), HviSat01-2531 produced strong hybridization signals in five chromosome pairs in the first and was restricted to a single sm pair at an interstitial position in the last. The HviSat13-730 was restricted to the secondary NOR constriction in a single pair in both species. The HviSat08-4011 followed the pattern for most other species, being accumulated in the telomeric region of the long arms of a submetacentric pair in both species. While HviSat05-177 was found in a single pair of *H. dissidens*, four pairs were probed in *H. guianensis*. Exclusively in *H. dissidens*, a scattered pattern was observed for HviSat03-997, and a single pair was labeled with HviSat04-2959, since no visible signals were found in *H. guianensis* for both satDNAs. Both HviSat18-1068 and HviSat02-2707 do not produce visible FISH signals. Figure 4 highlights *H. villasboas* satellite sequences that were hybridized to sex chromosomes in distinct selected *Harttia* species were schematized according to their phylogenetic relationships by Covain et al.^70^.

When hybridized with *H. punctata* chromosomes (Fig. 3e, f), the HviSatDNAs produced unexpected results. The sex chromosomes of this species accumulate several sequences, including HviSat08-4011 in a large pericentromeric block on X_1_, HviSat13-730 at the secondary constriction of X_2_, HviSat04-2959 also in a large pericentromeric block on X_1_, a small pericentromeric signal on X_2_ and Y, and HviSat05-177 at the centromere of X_1_ and X_2_, in addition to telomeric signals on X_2_ and in the Y, the last restricted to long-arms. HviSat04-2959 was also found in a small metacentric autosome pair and HviSat05-177 in the short arms of six autosomes. HviSat01-2531 was restricted to a large sm chromosome pair in an interstitial position on long arms, while HviSat02-2707 produced small signals in the pericentromeric region of a few chromosome pairs. The species *Harttia* sp. 3 (Fig. 3e, f) produced similar patterns to *H. villasboas* and *H. rondoni*. The at01-2531 was found in three chromosome pairs in the pericentromeric region, while HviSat03-997 was scattered in almost all chromosomes. HviSat18-1068 and HviSat13-730 were found in the secondary constriction of the first chromosome pair in the pericentromeric region. On the other hand, HviSat02-2707 was found slightly hybridized to some chromosome pairs, but only in one of the homologs of the first chromosome pair, all in the pericentromeric position. Both HviSat04-2959 and HviSat05-177 followed the pattern of a single chromosome pair hybridized with each probe, while HviSat08-4011 was found in the long arms of a submetacentric large pair.

### Comparative satellitomics

19 of the 21 *H. villasboas* satDNAs have a homologous counterpart in one, two, or three other *Harttia* species (Table 2). Table 2 reflects that there are conserved satellites in all four species analyzed (e.g., the homologs of HviSat01-2531, HviSat04-2959 or HviSat05-177, among others). However, there are lineage-specific satellites: two satDNAs, HviSat17-49 and HviSat20-47, appear to be specific to *H. villasboas*, six satDNAs appear to be specific to *H. rondoni*, 1 to *H. duriventris* and 4 to *H. punctata*. In this context, we have found some satellites that are specific to the *H. villasboas-H. rondoni* lineage: HviSat08-4011, HviSat16-2480, HviSat19-589, and HviSat21-575 have homologues only in *H. rondoni*, the closest relative to *H villasboas*. But this table also reflects the saltational process of evolution of some satellites that form the satellitomes of these species since there are satDNAs shared by two or more species for which the pattern of sharing does not conform to the phylogeny of Fig. 1: among several others, one example is HviSat02-2702 for which we have found homologs in *H. duriventris* and *H. punctata* but not in *H. rondoni*. These data reflect differential amplifications of these satellites in different species at different evolutionary times.

**Table 2:** Homologous satDNA families of *Harttia*

Species			
*H. villasboas*	*H. rondoni*	*H. duriventris*	*H. punctata*
HviSat01-2531	HroSat02-1662	HduSat03-2143	HpuSat12-1973
	HroSat03-372 HroSat05-515		
HviSat02-2707		HduSat01-2707	HpuSat04-2707
HviSat03-997			HpuSat05-1068
HviSat04-2959	HroSat09-2961	HduSat08-1754	HpuSat07-2750
HviSat05-177	HroSat16-177	HduSat06-177	HpuSat02-177
HviSat06-200		HduSat04-31	
HviSat07-93	HroSat01-93	HduSat05-93	HpuSat06-105
HviSat08-4011	HroSat14-4165		
HviSat09-815	HroSat19-815	HduSat11-815	HpuSat10-781
HviSat10-1666	HroSat06-1634	HduSat12-1670	HpuSat14-503
HviSat11-1338	HroSat13-1440	HduSat10-1449	HpuSat08-1546
HviSat12-144		HduSat13-144	HpuSat03-144
HviSat13-730	HroSat04-920	HduSat09-771	
	HroSat12-144	HduSat16-144	
HviSat18-1068	HroSat11-534	HduSat15-534	
HviSat14-347	HroSat08-347	HduSat02-347	
HviSat15-32		HduSat14-32	HpuSat11-32
HviSat16-2480	HroSat17-2481		
HviSat19-589	HroSat18-1194		
HviSat21-575	HroSat23-576		
	HroSat10-21	HduSat07-21	HpuSat01-21
	HroSat20-45	HduSat18-45	

Table 3 shows the genetic distances between the satDNAs of *H. villasboas* and the corresponding homologous satellites (i.e. the similarity between the homologous sequences of satDNAs, measured by the alignment of the sequences). In general, the greater the phylogenetic distance, the greater the genetic distance between homologous satellites. This is especially true in comparisons with *H. punctata*. But Table 3 also shows that there are highly accelerated or slowed rates of change for some satDNAs lineages, with genetic distances that are not consistent with phylogenetic proximity in some comparisons. For example, the homologous HviSat01-2531 in *H. rondoni* or the homologous HviSat04-2959 in *H. duriventris* show greater divergence from *H. villasboas* than homologs of phylogenetically more distant species. The table, in general, shows differential divergence patterns between different satellites and between different lineages. Taking as a reference the only node in which we have a clear dating to compare two species, Supplementary Table 9 shows CTR for each satDNA assuming a divergence time of 17.5 my between *H. villasboas* and *H. punctata* (Fig. 1), demonstrating very different rates of sequence change in different satDNAs.

**Table 3:** A comparison of the genetic distances between the homologous satDNAs of *Harttia villasboas* and the other three species (*H. rondoni*, *H. duriventris*, and *H. punctata*)

	Homologous satDNA		
*Harttia villasboas*	*Harttia rondoni*	*Harttia duriventris*	*Harttia punctata*
**HviSat01-2531**	0.081 (**1**)	0.023 (**2**)	0.189 (**3**)
	0.068 (**4**) 0.198 (**5**)		
	0.128 (**6**)		
**HviSat02-2707**		0.004 (**2**)	0.025 (**3**)
		0.023 (**5**)	
**HviSat03-997**			0.296 **(3**)
**HviSat04-2959**	0.018 (**1**)	0.390 (**2**)	0.180 (**3**)
	0.393 (**4**) 0.498 (**5**)		
	0.156 (**6**)		
**HviSat05-177**	0.006 (**1**)	0.006 (**2**)	0.163 (**3**)
	0.000 (**4**) 0.156 (**5**)		
		0.156 (**6**)	
**HviSat06-200**		0.067 (**2**)	
**HviSat07-93**	0.044 (**1**)	0.116 (**2**)	0.316 (**3**)
	0.142 (**4**) 0.350 (**5**)		
		0.367 (**6**)	
**HviSat08-4011**	0.016 (**1**)		
**HviSat09-815**	0.030 (**1**)	0.030 (**2**)	0.271 (**3**)
	0.043 (**4**) 0.280 (**5**)		
		0.275 (**6**)	
**HviSat10-1666**	0.016 (**1**)	0.023 (**2**)	0.498 (**3**)
	0.020 (**4**) 0.480 (**5**)		
		0.471 (**6**)	
**HviSat11-1338**	0.070 (**1**)	0.065 (**2**)	0.771 (**3**)
	0.025 (**4**) 0.775 (**5**)		
		0.768 (**6**)	
**HviSat12-144**		0.007 (**2**)	0.007 (**3**)
		0.000 (**5**)	
**HviSat13-730**	0.092 (**1**)	0.109 (**2**)	
	0.048 (**4**)		
**HviSat14-347**	0.012 (**1**)	0.032 (**2**)	
	0.038 (**4**)		
**HviSat15-32**		0.000 (**2**)	0.000 (**3**)
		0.000 (**5**)	
**HviSat16-2480**	0.015 (**1**)		
**HviSat18-1068**	0.038 (**1**)	0.067 (**2**)	
	0.061 (**4**)		
**HviSat19-589**	0.107 (**1**)		
**HviSat21-575**	0.007 (**1**)		

Genetic distances were calculated from a consensus sequence built from all variants and are displayed according the following species pair (**1**) Hvi-Hro, (**2**) Hvi-Hdu, (**3**) Hvi-Hpu, (**4**) Hro-Hdu, (**5**) Hdu-Hpu, (**6**) Hro-Hpu.

### Repeat landscapes of Harttia satDNAs

Supplementary Fig. 6 displays repeat landscape (RL) plots representing, for several *Harttia* satDNAs, abundance (y-axis) and divergence (x-axis) to the consensus sequence built for each satDNA repeat unit. satDNA degenerates through point mutation (increasing divergence) and homogenizes through amplification (decreasing divergence). Then, it is assumed that peaks at lower divergence values in the repeat landscape profiles are the product of recent amplifications, whereas those at higher divergence values are probably older variants degenerated by the accumulation of mutations. Following this argument, we have analyzed the RL plots of satDNAs found in the sex chromosomes of *Harttia* species. Thus, the satellite HviSat08-4011 and its counterpart HroSat14-4165 show a unique amplification peak at 0% divergence. A paired samples t-test found no significant differences between both satDNA repeat landscape profiles (t = −0.83, df = 40, *p* = 0.2). Then, although the graph shows sequence variants of these satellites with divergences of up to 40%, the amplification that resulted in the current status of this satellite in the *H. villasboas-H. rondoni* lineage must have been very recent after the lineage split that led to *H. duriventris*. In the case of the HviSat13-730 and HviSat18-1068 satellites and their counterparts, several peaks are observed that could reflect different, recent (the highest peaks are at 0% divergence) amplification waves of these satellites at different times in the distinct species. Paired samples t-test found significant differences for all pair comparisons between HviSat13-730 and HviSat18-1068 and their counterparts (Supplementary Table 8). The RLs of HviSat04-2959 and HviSat05-177, and their counterparts, show several peaks. Differences were significant in all pair comparisons between the repeat landscapes profiles, except for HviSat04-2959/HroSat09-2961 (Supplementary Table 8). The greatest peaks of the satellites HpuSat02-177 and HpuSat07-2750 stand out, which could be related to their incorporation and amplification in the sex chromosomes of *H. punctata* (these satellites are in autosomes in the other species) possibly after the separation of the lineages (17.5 mya; Fig. 1) since the divergence of 2% of these peaks would correspond to a divergence time of about 8 my (Table 3). Finally, the rest of the RL plots in Supplementary Fig. 6 represent examples of four autosomal satellites (HviSat01-2531, HviSat03-997, HviSat14-347, and HviSat19-589, and their counterparts). As shown, all these satellites show several peaks that could reflect different expansion waves, most of which are very old (there are divergence peaks of more than 20%), differentiated in time between the compared species (see also Supplementary Table 8).

### The complex nature of HviSat08-4011

As indicated, homologous satellites to the satellite HviSat08-4011 and its counterpart HroSat14-4165 in *H. rondoni* were not isolated from the genomes of *H. duriventris* and *H. punctata*. However, FISH with probes derived from HviSat08-4011 detected large blocks of hybridization of this satellite on chromosome X_1_ of both species, in addition to an autosomal locus (only in *H. duriventris*), which contrasts with the unique autosomal location of this satellite in *H. villasboas* and *H. rondoni* (as well as in other species lacking sex-chromosomes) (Fig. 4). BLASTn and BLASTx searches of HviSat08-4011 reveal a complex satDNA composed of at least two differentiated parts (Supplementary Fig. 5A). The largest part (about 2300 bp) is a sequence with no homology to any other known sequence. However, about 1700 bp of HviSat08-4011 shows homology and high identity (~80%) with the zona pellucida sperm-binding protein 4-like (*ZP4*) gene of diverse species of the orders Siluriformes (such as *Ictalurus, Silurus*, *Neoarius* and *Tachysurus*) and Characiformes (such as *Colossoma*, *Pygocentrus* and *Astyanax*). As Supplementary Fig. 5A shows, the homologous parts are discontinuous, separated by intervening unknown sequences. However, the homologous parts align continuously with ~90% of the translated sequence of the gene in those other species, with small discrepancies at the boundaries.

The analogous region of the *ZP4* gene is where the primers utilized in our initial FISH strategy were constructed (Supplementary Fig. 5A, F1R1). Two additional primer pairings have been put to the test. One is located in the *ZP4* non-homologous region (Supplementary Fig. 5A, F2R2), while the other is made up of a reverse primer inside the *ZP4* homologous region and a forward primer outside of it (Supplementary Fig. 5A, F3R3). In the first case, the results differ from those initially found (Supplementary Table 10 and Supplementary Fig. 5B): a) the two satellite parts (*ZP4*-homologous and non-*ZP4*-homologous) hybridize only on one autosome in *H. rondoni* and *H. villasboas*; b) the *ZP4*-homologous satellite part hybridizes on X_1_ and one autosome of *H. duriventris* but the satellite part not homologous to *ZP4* hybridizes only on the autosome; c) the satellite part homologous to *ZP4* hybridizes only on X_1_ of *H. punctata* and the satellite part not homologous to *ZP4* does not hybridize on any chromosome. In the second case, with the F3R3-HviSat08-4011 primers, only *H. villasboas* and *H. rondoni* produced visible fragments in the agarose gel (Supplementary Table 10 and Supplementary Fig. 5B). Those last PCR product amplifications were then sequenced to check the composition of the amplified fragments, and they fully coincided with the expected region.

We have searched for HviSat08-4011 among the Illumina reads of *H. duriventris* and *H. punctata* using the script mapping_blat_gs.py (see Materials and Methods) but we obtained no results other than partial sequences with homology to the portion homologous to *ZP4* (mostly) and some with homology to the portion not homologous to *ZP4*. We then mapped and quantified the raw reads of both species against both regions of HviSat08-4011 (repeat_masker_run_big.py) and obtained FISH-compatible results. Specifically, while this satDNA represents 0.05% and 0.01% of the genome of *H. villasboas* and *H. rondoni*, respectively (Supplementary Data 1), the part homologous to *ZP4* accounts for 0.1% of the genome of *H. punctata* while the non-homologous part accounts for 0.03% of its genome, these percentages being 0.03% in both cases in *H. duriventris*. Supplementary Fig. 5C shows repeat landscape plots representing, for *H. villasboas*, *H. rondoni*, *H. duriventris*, and *H. punctata*, abundance (y-axis) and divergence (x-axis) with respect to a consensus sequence built for each part of the HviSat08-4011 repeat unit. The graphs show different evolutionary trajectories for each part (homologous to *ZP4* and non-homologous to *ZP4*) in *H. duriventris* and *H. punctata*. Thus, we can observe in *H. duriventris* several amplification peaks with divergences between 15% and 6% for the *ZP4* gene homologous part, not found for the non-homologous part, in addition to a recent peak (0% divergence) that in this case does coincide with the major peak found for the non-homologous part. In *H. punctata* there are several major peaks at a divergence between 4 and 10% for sequences homologous to the *ZP4* gene, while the non-homologous to *ZP4* gene part shows smaller amplification peaks at divergences higher than 16%. Differences between the profiles of the two parts in *H. punctata*, in addition to revealing amplification events that are different in evolutionary times as in the case of *H. duriventris*, are statistically significant (t = −2.423, df = 40, *p* = 0.01). Furthermore, paired samples t-tests between each species and H. punctata found significant differences for the landscape profiles of the ZP-4 gene homologous part (p < 0.05).

### Relationship of Harttia satDNAs with transposable elements (TEs)

Supplementary Tables 4–7 show the relationships between transposable elements (TEs) and the various groups of *Harttia* homologous satellites. The most remarkable results are: a) out of the several satellites found in the sex chromosomes in this study, only the satellite HviSat04-2959, and its homologs in the rest of the species, is related to this type of elements; b) most of the satellites are complex ones composed of different parts in which sequences derived from SINE and/or LINE elements and tRNA genes (and satDNAs derived from tRNAs) are intermingled with intervening sequences; c) HviSat02-2707 and its homolog are connected to Penelope elements, whereas HduSat17-150, which has no homologs in other species, is related to Helitron elements; d) HviSat06-200, but not its shorter homologous HduSat04-31, is analogous to a mosaic zebrafish satDNA.

## Discussion

Our results show that the complex history of chromosomal evolution in *Harttia* is also reflected in its satDNA content. We demonstrated the occurrence of distinct profiles of satDNAs on homologous sex chromosomes, suggesting that these repeats diverged fast, recently, and independently in each species. SatDNA sequences are abundant on sex chromosomes and may play an important role in gene regulation and dosage compensation, resulting in hybrid incompatibilities^36,71^. Moreover, our data demonstrated that in other *Harttia* without heteromorphic sex chromosomes, a satDNA (HviSat08-4011) is amplified in the same linkage group that creates the sex chromosomes seen in the four target species of this study, suggesting an ancestral sex chromosome system, which will be discussed below.

### Evolution of satellite DNAs in Harttia genomes

A large number of satDNA families are shared among the four *Harttia* species investigated in this study. This finding supports the library hypothesis^72^, which has already been reported for other groups, including fishes^69,73,74^, mammals^75,76^, reptiles^77^, insects^78,79^, and plants^80–82^. The library hypothesis states that species possess a repertoire of satellite DNA (satDNA) families, which exhibit variations in both the number of copies and their arrangement within the genome^72^. As seen in Table 2, which is the result of differential amplifications of each satDNA in different species at different evolutionary times, thus supports this hypothesis. Roughly, genetic distances between homologous satellites agree with the phylogenetic relationships shown in Fig. 1. Therefore, the distances between *H. villasboas* and *H. rondoni* are consistently the shortest, indicating that these two species are the closest in phylogenetic proximity. The primary differences consistently occurred between *H. punctata* and any other species, indicating that this lineage split from the lineage that originated the clade consisting of the other three species a long time ago (Fig. 1). However, in agreement with the library hypothesis, differential amplifications lead to accelerated rates of change in different lineages as disparate genetic distances that do not fit with phylogenetic relationships are observed (Table 3). In this context, the repeat landscapes indicated peaks in both higher and lower divergence values, probably due to the presence of older variants degenerated by the accumulation of mutations in addition to recent amplification and homogenization events (Supplementary Fig. 6).

Satellite DNAs (satDNAs) exhibit variation in their location on *Harttia* chromosomes, ranging from being scattered in a few places throughout the chromosome to being mostly confined to either the pericentromeric or telomeric regions (Figs. 2 and 3), the preferential places of clusterization of satDNAs^36,83^. It has been proposed that satDNAs may play a significant role in the structure and operation of both centromeric and telomeric regions since such distribution lines up within all eukaryotes^84,85^. In terms of size, there is a prevalence of long satDNAs (>100 bp), which is consistent with what has been seen in the related species *H. carvalhoi*^74^.

Some satDNAs of *Harttia* also share the position with the 18S rDNA secondary constriction, following HviSat13-730 and HviSat18-1068 associated with the secondary NOR constriction of most species, with the last being especially highlighted in *H. villasboas*, *H. rondoni*, *H. duriventris*, and *Harttia* sp. 3 (Figs. 1–4). All four species that harbor the multiple X_1_X_2_Y sex chromosome system demonstrated some ribosomal DNA loci in both X and Y chromosomes. While *H. punctata*^86^ carries both 5S (X_1_ and Y) and 18S rDNA (X_2_), only the 18S rDNA is found in the sex chromosomes of *H. duriventris* and *H. villasboas* (X_2_ and Y)^87^. The sister species with the homologous simple XY system (*H. rondoni*^87^) has the 18S rDNA locus in both sex chromosomes. Ribosomal sequences were suggested to increase the instability of genomes due to their high transcriptional activity, which can facilitate double-strand breaks^88,89^, as suggested for other *Harttia* species^90^, and also other loricariids such as *Ancistrus*^91^ and *Rineloricaria*^92,93^. In plants, numerous satDNAs arise from the intergenic spacer of the ribosomal locus^94,95^, which was not observed in *Harttia* genomes. Since the rDNAs located in the sex chromosomes of *Harttia* were suggested to surround or be inserted in an evolutionary breakpoint region that is reused in the karyotypic diversification of the genus^34^, both HviSat13-730 and HviSat18-1068 may likely have contributed to the genomic instability that led to the fission process the originates the multiple sex chromosomes herein investigated. SatDNAs are also associated with the formation of chromocenters and micronuclei (reviewed in ref. ^96^), which can trigger DNA breakage and incompatibilities between populations, leading to reproductively isolated populations^97^.

Transposable elements (TEs) are known to contribute to genome expansion mechanisms primarily through retrotransposition, but also by creating new tandem repeats^98^ by partial TE duplication followed by uneven crossing-over^99^, or by transposase-induced breaks and repair mechanisms^100^. In *Harttia*, these elements have an intricate relation with the formation of satDNAs, as demonstrated by complex sequences composed of distinct parts in which motifs derived from short-interspersed elements (SINE) and/or long interspersed elements (LINE) and tRNA genes. For example, HviSat02-2707 and its homologs in other species are related to Penelope elements, a class of repetitive elements widely distributed in fish genomes, seen in medaka, pufferfishes, and cichlids^101^. Penelope-like elements (PLE) are predominantly found in animal genomes, with occasional losses in some lineages^102,103^. On the other hand, the *H. duriventris-*specific satellite HduSat17-150 is related to Helitron, a group of TEs that conserves sequences at ends but with satDNA-like central tandem-repeated motifs^104^, including real satDNAs embedded in such central position in Bivalves^105,106^. This demonstrates that the evolutionary history of satDNA sequences is also related to TEs, reflecting the complex history of chromosomal rearrangements that occurred in the genus. However, such TEs do not seem to participate in sex chromosome differentiation, as discussed in the next section. Most satDNAs in this genus presented rates of divergence between homologous sequences that vary according to the phylogenetic relationships, but repeat landscapes demonstrated species-specific amplification and homogenization events, especially in the repeats found in sex chromosomes. Such patterns indicate a contingent evolution scenario for the satDNAs of *Harttia*, as also seen in the repetitive content of grasshoppers^107^.

### The role of satDNAs in the evolution of Harttia´s sex chromosomes

Although all *Harttia* species from Clade III share homologous sex chromosomes, as indicated by chromosome painting studies^11^, each demonstrated a distinct composition of satDNAs, indicating independent evolution of these sequences in each lineage. Sex chromosome differentiation by independent accumulation of satDNAs in a recent evolutionary time may reflect the neo origin of such systems, as also seen in other fish with recently originated sex chromosomes, such as *Megaleporinus elongatus* and *Nothobranchius* spp^41,43,47^. In *Harttia*, the discrepancy in satDNA profiles can be observed, especially in sequences that do not follow a phylogenetic arrangement, for example, HviSat08-4011, which was probably amplified in *H. duriventris* and *H. punctata* after their split, besides HviSat13-730 and HviSat18-1068, which we will discuss below.

The sex chromosomes of *H. villasboas* harbor only two satDNAs: HviSat13-730 and HviSat18-1068. These satellites share a minor part of their repeat sequence (about 6%) with 84% similarity, suggesting an ancient common origin. However, both satDNAs and their homologous counterparts in each species, have signals of recent amplifications in *H. villasboas*, *H. rondoni*, and *H. duriventris*, in addition to independent events of previous amplifications (Supplementary Fig. 6). For HviSat18-1068 and its homologs HroSat11-534 and Hdu15-534, which are smaller in size, a duplication process in *H. villasboas* was responsible for their difference in size. This satellite is presented in the secondary constriction formed by the 18S rDNA, where events of unequal crossing-over were proposed as the main driver for differences in the accumulation of this ribosomal sequence between the homologs^87^. Since these satellites are not linked to TEs, unequal recombination may have caused the duplication, as described for other satDNAs in rice and monkeys^108,109^. However, rolling circle replication may also be responsible for the duplication of such repetitive sequences^110^. Regarding HviSat13-730 and its homologs HroSat04-920 and HduSat09-771, which showed positive FISH signals in all four species, we could not identify homolog sequences in *H. punctata* genome. In this species, the FISH signal is restricted to the X_2_ chromosome and lacking in the Y, matching the 18S rDNA accumulation pattern^86^, suggesting that the fission event that created the multiple sex chromosomes may also explain this variation^11^.

On the other hand, out of the several satellites found in *H. punctata* sex chromosomes (see Fig. 3e) only HviSat04-2959 and its homologs (HroSat09-2961, HduSat08-1754, and HpuSat07-2750), are related to TEs. This satellite has partial homology with LINE-Rex-Babar and Tc/Mariner elements (see Supplementary Tables 4–7), which are usually inactivated or degenerated in fish genomes but can accumulate mutations at neutral rates until losing their molecular identity^111–114^. Both elements have been identified in related Loricariidae species, but as dispersed sequences without any accumulation on a specific chromosome pair^115^. In catfishes, Tc1-Mariner elements can be found associated with rDNA loci variation, suggesting activity in the transposition system^116^. Both satellites HviSat04-2959 and HviSat05-177 and their homologous counterparts, located on sex chromosomes in *H. punctata* but on autosomes in the rest of the species, have been expanding over more than ~20 million years in the four species. However, there has been a significant increase in the number of these satellites in *H. punctata*, which aligns with the observation of extra loci on the X_2_ chromosome of this species. TEs are less likely to build up on young X chromosomes in fishes compared to autosomes^117^, and this might be the reason for the scarcity of TE-derived satellite sequences in the sex chromosomes of *Harttia*.

The most intriguing relation between a satDNA sequence and sex chromosomes in the genus was observed with HviSat08-4011, which has a partial homology with the *ZP4* gene responsible for the formation of the egg envelope. Zona pellucida glycoproteins are generated in the ovary or liver by ZP genes^118^. The HviSat08-4011 is consistently mapped to autosomes and the linkage group is mapped by the whole chromosome painting probe HPU-X1 across the *Harttia* phylogeny (Supplementary Fig. 6), except in *H. villasboas* and *H. rondoni*, where only autosomal FISH signals were detected. Repeat landscapes show a symmetrical graphical pattern for both homologous and non-homologous *ZP4* satDNAs in *H. villasboas* and *H. rondoni*, which would indicate a single autosomal satellite recently originated in both species. However, the graph in *H. duriventris* would be compatible with an older amplification of a satellite formed from partial sequences of the *ZP4* gene in X_1_, possibly in addition to a more recent amplification of the composite satellite (composed of the two parts, the homologous and the non-homologous parts to *ZP4* gene) in autosomes as revealed by FISH. This is more evident in *H. punctata* where there are several major peaks representing the most abundant set of sequences homologous to the *ZP4* gene, at a divergence between 4 and 10%, compatible with several waves of amplification of a *ZP4*-derived satDNA on chromosome X_1_. The non-homologous to *ZP4* gene part, not detected by FISH in *H. punctata*, shows relatively smaller amplification peaks at divergences higher than 16%. The ZP genes in vertebrates undergo frequent gene duplication and loss events, suggesting that they are reproductive genes that evolve rapidly^119^. This rapid evolution may enable them to adapt to various ecological environments^120^ and serve as barriers to fertilization^121,122^, making them potential targets for natural selection. A fertilization-related gene in the same linkage group as the ancestral of the three *Harttia* clades may imply an ancestral sex chromosome system. Indeed, the linkage group mentioned is exclusively recruited as sex chromosomes in Clade III, which includes the species under investigation. This suggests a turnover event in the emergence of multiple XY_1_Y_2_ chromosomes in Clade II^11,34,35,74,93^.

The species with the most satDNAs accumulated in sex chromosomes (*H. punctata*) is also the oldest divergent in the clade (17.5 My), when compared to a much more recent diversification of *H. villasboas*, *H. rondoni*, and *H. duriventris* clade at 5.5 My^123^. However, each species revealed a distinct satDNA profile, with independent amplification and homogenization events occurring, suggesting an important role of these repetitive sequences in sex chromosome differentiation in a short evolutionary time, especially in recently originated sex chromosomes. Besides, satDNAs may have contributed to the speciation process in *Harttia*, since these sequences play important roles in chromosome pairing and segregation, indicating that the distinct profiles on homologous sex chromosomes may have contributed to reproductive isolation.

## Material and methods

### Specimens, chromosomal obtainment, and DNA sequencing

We collected adult samples of nine *Harttia* species, namely *H. dissidens*, *H. duriventris*, *H. guianensis*, *H. kronei*, *H. intermontana*, *H. punctata*, *H. rondoni*, *H. villasboas*, and *Harttia* sp. 3 (a distinct karyomorph from Rio do Peixe, PA, Brazil, described in ref. ^124^ that is not assembled to any known species), in distinct localities inside the Brazilian territory according to Fig. 1 and Supplementary Table 11. We have complied with all relevant ethical regulations for animal use, as indicated by ARRIVE guidelines. For most experiments, *H. villasboas* was chosen as the main target for comparisons because it represents the species with a multiple sex chromosome system (X_1_X_2_Y) that is more closely related to *H. rondoni*, which harbors the simple XY that is considered to be the ancestral condition for the sex chromosomes in the genus. After anesthesia with clove oil (Eugenol diluted in 95% ethanol in a final concentration of 60 mg/L in water), as approved by the Ethics Committee on Animal Experimentation of the Universidade Federal de São Carlos (Process number CEUA 7994170423), chromosomes were obtained from the anterior kidney following the classical air-drying technique^125^. The liver and muscle tissues were stored at 4 °C in 100% ethanol for molecular analysis. Total DNA was extracted from these tissues using a silica spin-column-based kit (Cellco Biotech, São Carlos – SP, Brazil). The purified DNA of species with described sex chromosomes (i.e. *H. duriventris*, *H. rondoni*, *H. villasboas*, and *H. punctata*) was sequenced on BGISEQ-500 platform (BGI Genomics, Shenzhen, China), generating 2 ×150 bp short read sequences. For *H. villasboas* and *H. rondoni*, males and females were sequenced, while for *H. duriventris* and *H. punctata*, only male samples were targeted.

### Satellite DNA library, sequence analysis and primer design

The raw reads obtained after the shotgun sequencing were trimmed with Trimmomatic^126^ to select pair-end reads with Q > 20 for all nucleotides. Then, the catalogs of satellite DNAs (satellitome) of each analyzed species (i.e. *H. duriventris*, *H. rondoni*, *H. punctata*, and *H. villasboas*) were characterized by running several iterations of the TAREAN tool^127^, each followed by filtering out the reads with similarity to the identified satDNAs using DeconSeq^128^, until no satDNA was found. Subsequently, we performed a homology search with RepeatMasker^129^ to group the sequences into variants, families, and superfamilies, as suggested by Ruiz-Ruano et al.^130^. Abundance and divergence values of each satDNA were estimated by masking male and female genomic libraries against the catalogs of satDNAs with RepeatMasker^129^ with the publicly available script (https://github.com/fjruizruano/satminer/blob/master/repeat_masker_run_big.py), using 10,000,000 reads (2 × 5,000,000). Additionally, repeat landscapes were generated to estimate the average divergence of satDNAs considering the genetic distances between sequences based on the Kimura-2-parameter model using the script calcDivergenceFromAlign.pl of the RepeatMasker suite (i.e. the similarity between the homologous sequences of satDNAs, measured by the alignment of the sequences).

Based on the abundance, satellites were named using the first letter of the genus and the two first letters of the specific epithet (HviSat for *H. villasboas*, HroSat for *H. rondoni*, Hdu for *H. duriventris*, and Hpu for *H. punctata*). To detect sex-specific accumulated satDNAs, we selected those with an F/M ratio (quotient of satDNA female and male abundances) higher than 1.2.

We searched for homology between *Harttia* satellitomes with the rm_homology script^130^ that makes all-to-all alignments with RepeatMasker v4.0.5^129^. For correspondent satellites between species, we did pairwise alignments using ClustalX^131^, which were manually revised. Sequence divergence between satDNA families of each pairwise comparison was calculated following Kimura’s two-parameter method (K2P)^132^ using MEGA11^133^. A consensus turnover rate (CTR) was calculated using the CTR = K/2 T equation, where T = divergence time (see nodes in the phylogenetic tree of Fig. 1) between species and K = K2P distance^107^.

In the absence of a chromosome-scale genome assembly for the genus, the whole satellitome was BLAST-searched^134^ against NCBI nucleotide collection to check for the presence of conserved satDNAs. Putative open reading frames were prospected in the satellitomes using “orfipy”^135^ and Geneious 7.1.3, with a minimum size of 100 bp. Identified ORFs were translated to protein sequences using SMS^136^ and searched against the NCBI database using BLASTp^137^, following filtering for query cover and percentage identity >70%.

In addition, we searched Repbase^138^ for homologies with transposable elements with RepeatMasker^129^ with “no_low” and “no_is” options.

We searched for some satDNAs like HviSat08-4011 among the Illumina reads of species like *H. duriventris* and *H. punctata*, from which we could not isolate its homologous counterpart with TAREAN, using the publicly available script mapping_blat_gs.py (https://github.com/fjruizruano/ngs-protocols/blob/master/mapping_blat_gs.py). Raw reads of both species were mapped and quantified against every two parts described in HviSat08-4011 using the script repeat_masker_run_big.py, as described above.

Primers were designed for the eight most abundant of the total 21 satDNAs identified in *H. villasboas* using the abovementioned process. We selected *H. villasboas* for the construction of probes since this species has multiple X_1_X_2_Y and is more closely related to the XY-carrier species *H. rondoni* (when compared to *H. duriventris* and *H. punctata*), which allows us to infer the role of the rearrangements that occurred in the clade. The consensus sequences for each of those satDNAs were manually investigated to design PCR primers (Supplementary Table 2). Then, satDNAs were amplified by PCR using a starting denaturation step of 95 °C for 5 min, 30–35 cycles with 95 °C during 20 s, with 39.1–54.2 °C as annealing temperature during 40 s, 72 °C during 30 s, and a final extension step of 10 min (Supplementary Table 2). The resulting PCR products were checked on a 1% agarose gel to confirm the typical ladder pattern characteristic of satDNAs (Supplementary Fig. 1). Sequences were deposited on Genbank (Access numbers: OR827473-OR827555).

### Fluorescence in situ hybridization

According to the manufacturer’s instructions, the selected PCR-amplified satellites of *H. villasboas* were directly labeled with Atto550-dUTP or Atto488-dUTP by Nick-Translation (Jena Biosciences, Jena, Germany). Probes were composed of 100 ng of each labeled satellite DNA plus 50% formamide, 2× SSC, 10% SDS, 10% dextran sulfate, and Denhardt’s buffer at pH 7.0 in a total volume of 20 µl. FISH experiments were conducted firstly in *H. villasboas* slides with every single probe, then in double-FISH for sister species *H. rondoni*, *H. duriventris*, and *H. punctata*, in addition to *H. dissidens*, *H. guianensis*, and *Harttia* sp. 3. The sex chromosomes were identified using their morphological characteristics, as established in prior studies refs. ^87,124^. The following morphological traits were employed to discern the sex chromosomes: of both *H. villasboas* and *H. duriventris*, both X_2_ and Y possess a prominent secondary constriction owed to the 18S rDNA loci. In *H. rondoni*, the same applies to both the X and Y chromosomes. In *H. punctata*, the 18S rDNA loci is confined to the X_2_ chromosome, while the Y chromosome corresponds to the largest metacentric chromosome of its karyotype. Finally, the X_1_ in *H. villasboas*, *H. duriventris*, and *H. punctata* represents the largest acrocentric chromosome of their karyotypes. Following the results of satDNAs probed on sex chromosomes, we performed whole chromosome painting (WCP), together with a FISH mapping of these satellites, using probes derived from microdissection of the sex chromosomes of *H. punctata* (HPU-X1 and HPU-X2), as previously described in refs. ^11,34^. For the investigation of sex chromosome evolution, we included two species (*H. kronei* and *H. intermontana*) that carry the ancestral condition of the linkage groups that compose the multiple X_1_X_2_Y (i.e., both HPU-X1 and HPU-X2 located in synteny on the same chromosome)^34^. In these last two species, we mapped the HviSatDNAs that were found accumulated in the sex chromosomes of the four main targeted species and detected the ancestral linkage groups through WCP. Chromosomes were denatured in 70% Formamide/2 × SSC at 72 °C for 3 min, probes at 86 °C for 8 min, then cooled at 4 °C for 2 min before being applied to slides. The hybridization was carried out for 16 h in a dark, moist chamber at 37 °C. Following a 5-min post-hybridization wash with 1×SSC at 65 °C, next with a 4×SSC/Tween solution at room temperature. Finally, chromosomes were counterstained with DAPI mounted in Vectashield (Vector Laboratories, Burlingame, USA). For the WCP experiment, chromosomes were denatured as abovementioned, but the probes were denatured at 85 °C for 8 min, cooled at 4 °C for 2 min, and pre-hybridized with the blocking sequence (unlabeled C0t-1 DNA, following^139^) at 37 °C for 1 h. Hybridization occurred for 48 h overnight at 37 °C in a dark, moist chamber. The final wash was performed using the same procedure as described above.

### Statistics and reproducibility

To confirm the 2n number, karyotype structure, and FISH results, at least 30 metaphase spreads per individual were examined. Images were captured with CoolSNAP on an Axioplan II microscope (Carl Zeiss Jena GmbH, Germany) and processed with ISIS (MetaSystems Hard & Software GmbH, Altlussheim, Germany). The map was generated using QGIS 3.32 (Lima) with a Natural Earth package and riverine information from HydroRIVERS^140^. Statistical analysis (t-test) was performed in Microsoft Excel (Office 365, Microsoft).

## Supplementary information

Supplementary Information

Description of Additional Supplementary File

Supplementary Data 1

## Data Availability

Sequencing data that support the findings of this study have been deposited in Genbank, with their accession codes OR827473-OR827555 (http://www.ncbi.nlm.nih.gov/nuccore/). All other data is available from the corresponding author on reasonable request.
