# Supplementary material for: Independent evolution of satellite DNA sequences in homologous sex chromosomes of Neotropical armored catfish (Harttia)
Source: Commun Biol. 2025 Mar 30;8:524. doi: 10.1038/s42003-025-07891-6 (PMC11955569; doi:10.1038/s42003-025-07891-6)
Supplement: Supplementary file 1 — Supplementary Information [file 42003_2025_7891_MOESM1_ESM.pdf]

Supplementary Information

Independent evolution of satellite DNA sequences in homologous sex chromosomes of Neotropical armored catfishes (*Harttia*)

Francisco de M. C. Sassi[1], Manuel A. Garrido-Ramos[2], Rodrigo Zeni dos Santos[3], Ricardo Utsunomia[3], Tariq Ezaz[4], Geize A. Deon[1], Fábio Porto-Foresti[3], Thomas Liehr[5*], Marcelo de B. Cioffi[1]

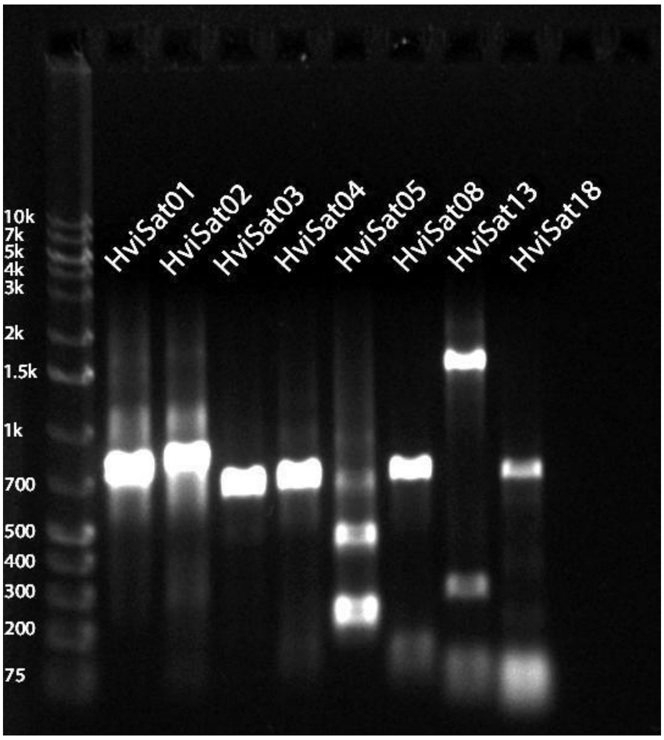

Supplementary Figure 1. Band patterns of satellites obtained by PCR with *Harttia villasboas* DNA as template in Agarose 1% gel with a 1kb ladder.

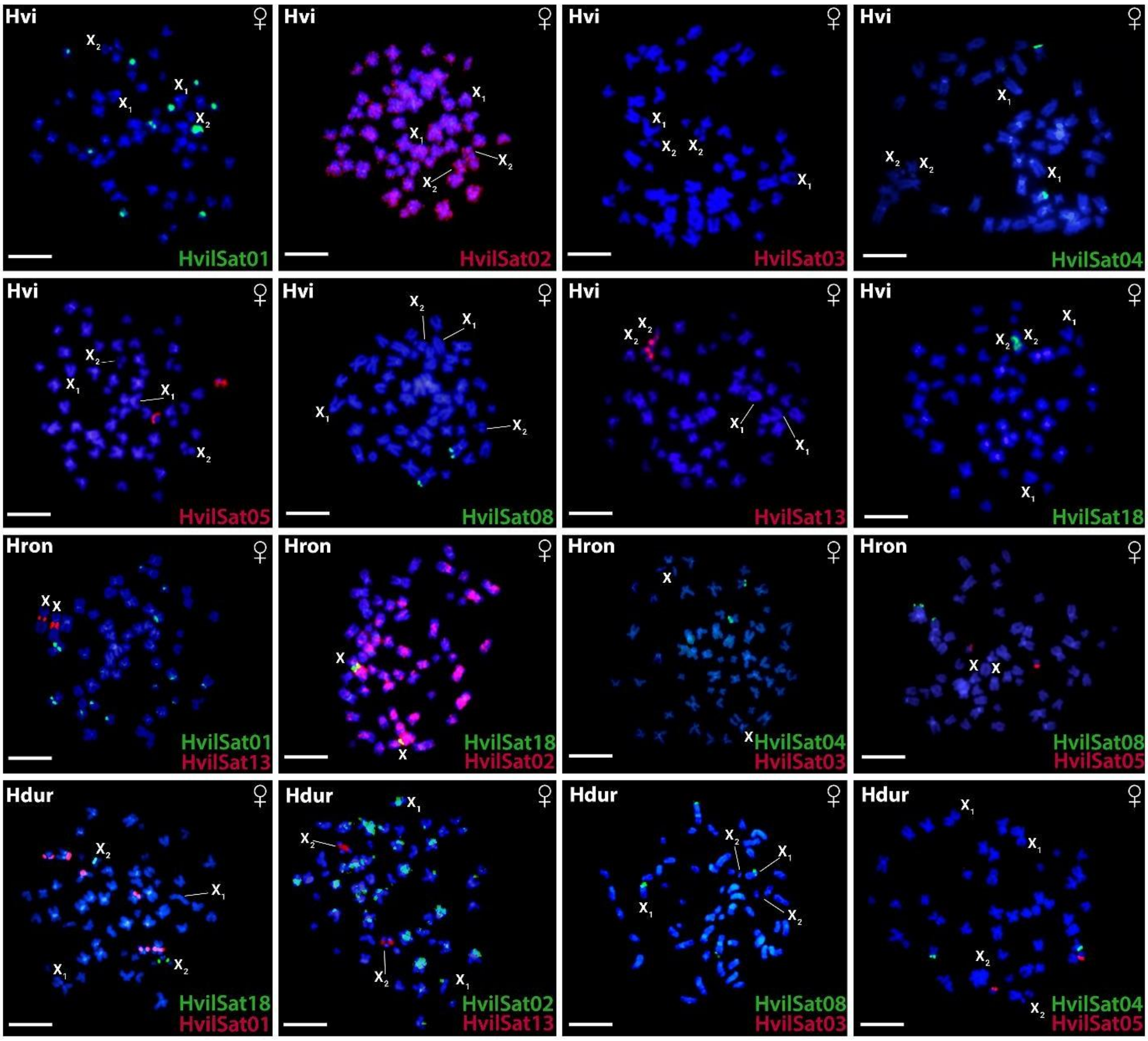

Supplementary Figure 2. FISH with *H. villasboas* satellitome probes on female metaphase chromosomes of *Harttia villasboas* (Hvi), *H. rondoni* (Hron), and *H. duriventris* (Hdur). Probes and their respective colors are indicated in the lower right corner and species in the upper left. Scale bar = 5 µm.

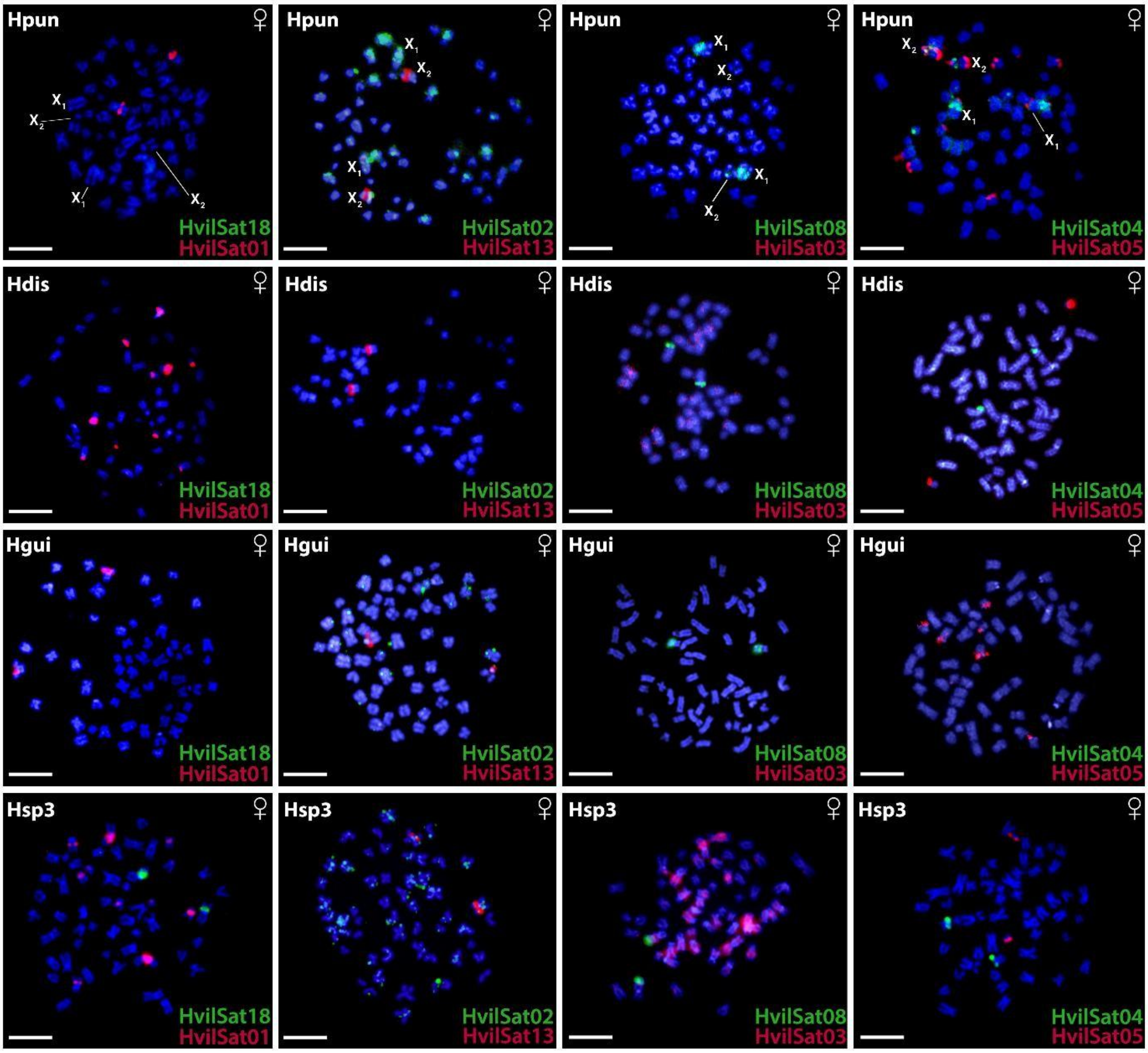

Supplementary Figure 3. FISH with *H. villasboas* satellitome probes on female metaphase chromosomes of *H. punctata* (Hpun), *H. dissidens* (Hdis), *H. guianensis* (Hgui), and *Harttia* sp. 3 (Hsp3). Probes and their respective colors are indicated in the lower right corner and species in the upper left. Scale bar = 5 µm.

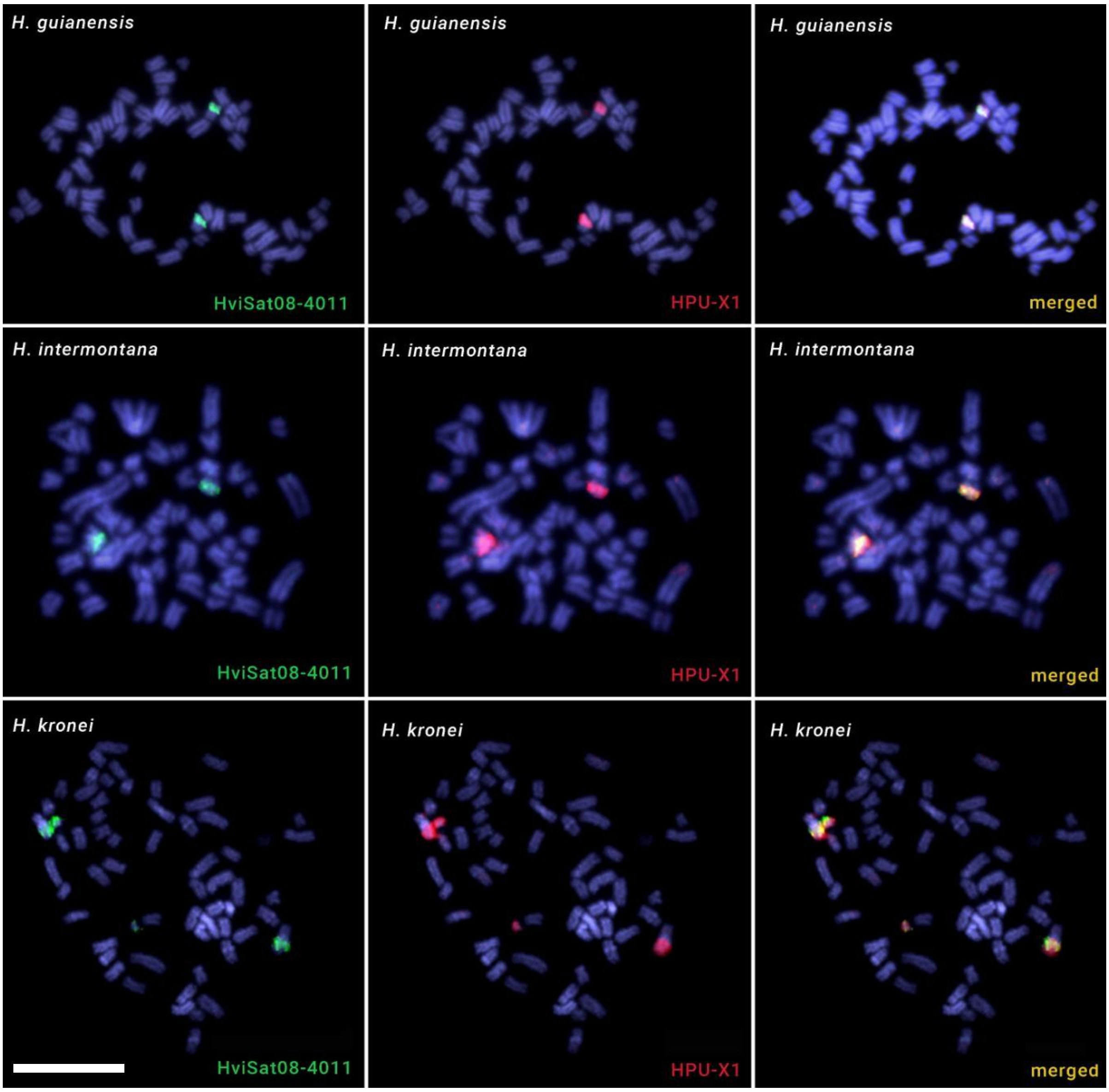

Supplementary Figure 4. FISH mapping of HviSat08-4011 (first column) and sequential WCP with HPU–X1 (second column) on species of *Harttia* without heteromorphic sex chromosomes derived from the linkage group that forms the multiple sex chromosomes in the four main target species of this study (lines). The third column correspond to both hybridizations merged in a single image to demonstrate the congruence in the position of both mapped probes. Scale bar = 10 µm.

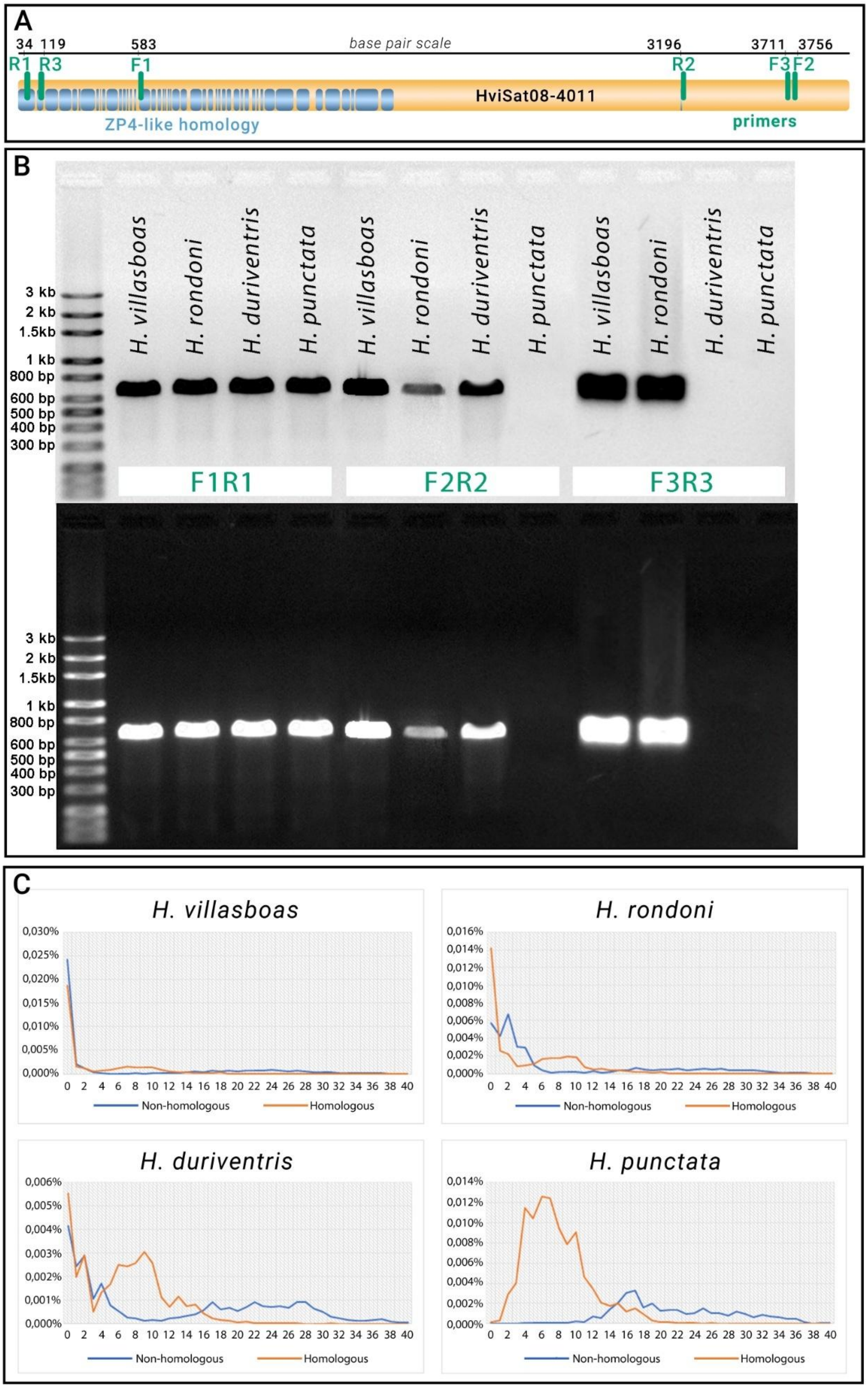

Supplementary Figure 5. Composition, distribution and evolution of HviSat08-4011. a) Structure of HviSat08-4011, indicating the homologous regions with the ZP4-like gene (blue) and the three sets of primers (F1R1, F2R2, and F3R3, in green) used for PCR, as demonstrated by b) the agarose-gel image, also showing the original gel image. c) Repeat landscape plots representing, for *H. villasboas*, *H. rondoni*, *H. duriventris*, and *H. punctata*, abundance (y-axis) and divergence (x-axis) to a consensus sequence built for each part of the HviSat08-4011 repeat unit, being blue lines for the non-homologous to ZP4 and orange for the homologous portion.

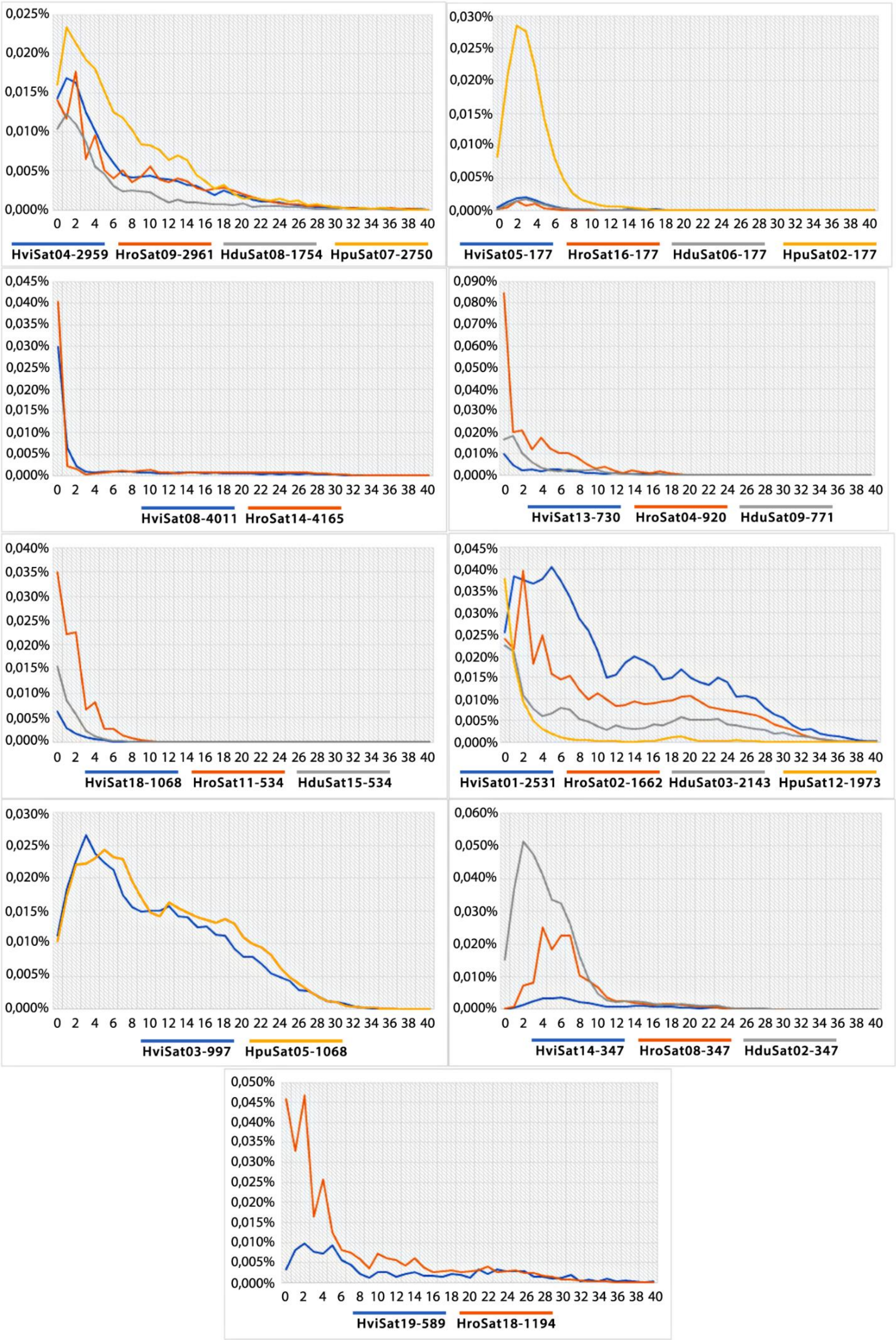

Supplementary Figure 6. Repeat landscape plots for *Harttia* satDNAs. Repeat landscape plots representing, for several *Harttia* satDNAs, abundance (y-axis) and divergence (x-axis) to a consensus sequence built for each satDNA repeat unit.

Supplementary Table 1. Distribution of sex chromosome systems in *Harttia* species. Cell colors reflect the homology of the sex chromosome systems. Data from [10,34] and [11,35].

Sex Chromosome System	Species	2n
$X_1X_1X_2X_2/X_1X_2Y$	*H. duriventris*	56♀/55♂
$X_1X_1X_2X_2/X_1X_2Y$	*H. villasboas*	56♀/55♂
$X_1X_1X_2X_2/X_1X_2Y$	*H. punctata*	58♀/57♂
XX/XY	*H. rondoni*	54♀♂
XX/XY_1Y_2	*H. carvalhoi*	52♀/53♂
XX/XY_1Y_2	*H. intermontana*	52♀/53♂
XX/XY_1Y_2	*Harttia* sp. 1	56♀/57♂
XX/XY	*H. torrenticola*	56♀♂

Supplementary Table 2. PCR conditions (primers, temperature, and concentration of template DNA) for the optimal amplification of satellite DNAs from *Harttia villasboas* genome.

Satellite	Primer F	Primer R	Annealing temperature range	[DNA] ng/µl
HviSat01-2531	5'GAAACCCAGGCAAAAGTTCA	5'CCTTCATACTCCCATGTAGCT	56 °C - 48 °C	100
HviSat02-2707	5'GGCTGAATTTTGCTGTGACAC	5'CCATAATGACGTCCCTCACC	56 °C - 48 °C	100
HviSat03-997	5'ACACTCATGACACTATCCCGT	5'CGGATTGTGCCCAGTAGCGTT	62.9 °C - 58 °C	100, 10, or 1
HviSat04-2959	5'TCGGCCAAAATACAGACTGCA	5'TTCCAGACGGCTGATGGCTTG	66 °C - 58 °C	100, 10 or 1
HviSat05-177	5'ACGAAACTCGCATATGTCGTT	5'ACACGTACGACTTCGGCTTA	61.4 °C - 55 °C	100 or 10
HviSat08-4011	5'ACAGAGTCCAAACTGAAGAGC	5'TCTTCTTCTTTTCCTCCACTG	62.9 °C - 58 °C	100, 10 or 1
HviSat13-730	5'CCACCTCTGCAAAAAACAAGGG	5'TTGATCAGAGGGGTGCCGTC	64.4 °C - 58 °C	100
HviSat18-1068	5'CGTTCCGATCGGGGAGGTCC	5'AGGCGCGTTCGGGGGACC	69°C - 62 °C	100

Supplementary Table 3. Putative Open Reading Frames (ORFs) identified in the four *Harttia* satellitomes, their BLASTp match (>70% Query Cover and >70% Percentage of Identity), and the start and stop bp in the satellite sequence.

Satellite	ORF	BLASTp	Start	Stop
HviSat02-2707	5	Uncharacterized protein from fish species	56	2456
HviSat04-2959	5	Hypothetical protein from *Prochilodus magdalenae*	1671	2001
HviSat04-2959	12	Putative nuclease HABI1 from *Trichomycterus rosablanca*, hypothetical protein from fish species	521	713
HviSat04-2959	14	Hypothetical protein from *Prochilodus magdalenae*	1901	2111
HviSat08-4011	23	ZP4-like from fish	168	384
HviSat08-4011	29	ZP4-like from fish	665	788
HviSat08-4011	34	ZP4-like from fish	808	1063
HviSat10-1666	1	Hypothetical protein from eukaryotes	21	141
HviSat10-1666	11	Mucin-1-like from *Cervus elaphus*, basic proline-rich protein-like from several mammals, dual specificity protein phosphatase 18 from *Kryptolebias marmoratus*	615	747
HroSat06-1634	2	Hypothetical protein from eukaryotes	124	244
HroSat09-2961	8	Hypothetical protein, putative RNA-directed DNA polymerase from transposon BS, F-actin-uncapping protein, RAC serine/threonine-protein kinase, all from fish	28	181
HroSat09-2961	13	Hypothetical protein from *Prochilodus magdalenae*	113	446
HroSat09-2961	17	Putative nuclease HABI1 from *Trichomycterus rosablanca*, hypothetical protein from *Prochilodus magdalenae*, uncharacterized protein from *Anguilla anguilla*, and putative RNA-directed DNA polymerase from transposon BS from *Labeo rohita*	1925	2117
HroSat14-4165	5	ZP4-like from fish	2652	2907
HroSat14-4165	13	ZP4-like from fish	3331	3547
HroSat14-4165	19	ZP4-like from fish	2927	3050
HroSat17-2481	3	Uncharacterized protein from fish, hypothetical protein from spider	1062	1209
HroSat23-576	3	Uncharacterized protein from vertebrates, hypothetical protein from vertebrates, NADH dehydrogenase iron-sulfur protein 7 from rabbit	327	444
HduSat01-2707	17	Uncharacterized Fish Protein	251	2651
HduSat08-1754	7	Putative nuclease HABI1 from *Trichomycterus rosablanca*, hypothetical protein from fish species	1184	1376
HduSat12-1670	10	Hypothetical protein from eukaryotes	340	460
HpuSat04-2707	12	Uncharacterized protein from fish species	277	2677
HpuSat07-2750	14	Hypothetical and uncharacterized fish proteins, zinc finger BED domain-containing protein 4 from *Anabarilius grahami*, putative RNA-directed DNA polymerase from transposon BS from *Labeo rohita*, transposon Tf2-8 polyprotein from *Labeo rohita*, hydrocephalus-inducing protein homolog from *Tachysurus fulvidraco*, gastrula zinc finger protein XlCGF28.1-like from *Silurus meridionalis*, LINE-1 retrotransposable element ORF2 protein from *Labeo rohita*	2432	2567
HpuSat07-2750	22	Hypothetical and uncharacterized fish proteins, putative RNA-directed DNA polymerase from transposon BS from *Labeo rohita*,	1537	1648

Satellite	ORF	BLASTp	Start	Stop
HviSat02-2707	5	Uncharacterized protein from fish species	56	2456
HviSat04-2959	5	Hypothetical protein from *Prochilodus magdalenae*	1671	2001
HviSat04-2959	12	Putative nuclease HABI1 from *Trichomycterus rosablanca*, hypothetical protein from fish species	521	713
HviSat04-2959	14	Hypothetical protein from *Prochilodus magdalenae*	1901	2111
HpuSat07-2750	27	adhesion G protein-coupled receptor E1-like from *Megalobrama amblycephala*, galactokinase isoform X1 from *Phyllopteryx taeniolatus*, teneurin-3-like from *Syngnathus typhle*, transposon Tf2-6 polyprotein from *Labeo rohita*, gastrula zinc finger protein XlCGF28.1-like from *Silurus meridionalis*, chloride channel protein 2b isoform X2 from *Entelurus aequoreus* putative RNA-directed DNA polymerase from transposon BS from *Labeo rohita*, hypothetical protein from *Hemibagrus guttatus*	1059	1230
HpuSat10-781	3	cilia- and flagella-associated protein 46 from *Pseudobagrus ichikawai*, hypothetical protein from *Channa argus* and *Tachysurus vachellii*	541	673
HpuSat12-1973	8	Uncharacterized protein from *Corvus cornix cornix*	1789	1936

Supplementary Tables 4-7. BLASTn results of Hvi (4), Hro (5), Hdu (6), and HpuSatDNAs (7), respectively, with positive match in repetitive sequences. Different columns indicate the following data: Smith-Waterman score of the match, complexity adjusted (SW); percentage of substitutions in matching region compared to the consensus (Div); percentage of bases opposite a gap in the query sequence (deleted bp); percentage of bases opposite a gap in the repeat consensus (inserted bp); position in query (begin-end); strand (transcript strand (+); complementary strand (C); matching repeat (Rep); repeat class/family (Class); percentage of matching satDNA sequence (%), and total percentage per satDNA (Total).

Supplementary Table 4

		Percentage			Position						
**Query**	**SW**	**Div**	**Del**	**Ins**	**Begin**	**End**	**Strand**	**Rep**	**Class**	**%**	**Total**
HviSat01-2531	354	20.0	0.0	0.0	78	137	C	tRNA-Val-GTG	tRNA	2,33	
HviSat01-2531	573	11.0	0.0	0.0	264	336	C	tRNA-Val-GTG	tRNA	2,84	
HviSat01-2531	405	18.3	0.0	0.0	1868	1938	C	tRNA-Asp-GAY	tRNA	2,77	
HviSat01-2531	420	19.4	0.0	0.0	2438	2509	C	tRNA-Asp-GAY	tRNA	2,81	10,75
HviSat02-2707	5446	29.9	0.0	2.6	1	2403	+	Penelope-5_XT	LINE/Penelope	88,73	
HviSat02-2707	355	24.7	0.0	2.0	2551	2699	+	Penelope-5_XT	LINE/Penelope	5,47	94,20
HviSat04-2959	4989	28.6	2.4	1.9	54	2055	+	Rex1-3_EL	LINE/Rex-Babar	67,62	
HviSat04-2959	338	27.1	9.3	0.0	2233	2302	+	tRNA-Ile-ATC	tRNA	2,33	
HviSat04-2959	591	9.6	5.1	0.0	2423	2495	C	tRNA-Ala-GCY	tRNA	2,43	
HviSat04-2959	302	26.8	0.0	1.1	2869	2955	+	Mariner-N4_SSa	DNA/TcMar-ISRm11	2,91	
HviSat04-2959	568	7.2	0.0	0.0	2891	2959	+	tRNA-Ile-ATT	tRNA	2,30	77,59
HviSat06-200	370	4.5	3.4	10.7	1	197	+	MOSAT_DR	SatDNA	98,00	98,00
HviSat09-815	597	4.3	0.0	0.0	1	70	+	tRNA-Gly-GGY	tRNA	8,47	
HviSat09-815	385	23.8	30.7	0.0	71	84	+	DNA-1_Gav	DNA	1,60	10,06
HviSat10-1666	646	4.9	0.0	0.0	46	127	+	Nimb-4_DR	LINE/I	4,86	
HviSat10-1666	595	10.8	0.0	0.0	633	715	C	tRNA-Leu-CTG	tRNA	4,92	
HviSat10-1666	349	17.1	13.6	0.0	1516	1585	C	Nimb-4_DR	LINE/I	4,14	13,93
HviSat11-1338	629	0.0	0.0	0.0	250	322	+	tRNA-3_DRe	tRNA	5,38	5,38
HviSat16-2480	628	4.2	0.0	0.0	12	83	+	tRNA-Gly-GGA	tRNA	2,86	
HviSat16-2480	275	26.1	0.0	0.0	519	587	+	tRNA-Lys-AAA	tRNA	2,74	
HviSat16-2480	600	5.6	0.0	0.0	748	819	C	tRNA-Trp-TGG	tRNA	2,86	
HviSat16-2480	397	19.0	0.0	3.7	1704	1785	+	tRNA-1_DRe	tRNA	3,27	
HviSat16-2480	630	6.7	0.0	0.0	1723	1797	+	tRNA-Lys-AAA	tRNA	2,98	
HviSat16-2480	249	30.3	5.7	1.0	1798	1822	+	Ves2_ML	SINE/tRNA	0,97	15,69
HviSat19-589	359	21.9	6.8	0.0	55	75	C	tRNA-3_DRe	tRNA	3,40	
HviSat19-589	363	20.8	0.0	0.0	76	147	C	tRNA-Arg-CGY	tRNA	12,09	
HviSat19-589	397	15.2	8.0	5.2	361	457	C	tRNA-3_DRe	tRNA	16,30	31,75
HviSat21-575	596	8.8	0.0	0.0	5	84	+	tRNA-1_DRe	tRNA	13,74	
HviSat21-575	665	0.0	0.0	0.0	320	393	C	tRNA-Asn-AAC	tRNA	12,70	26,43

Supplementary Table 5

Query	SW	Percentage Div	Del	Ins	Position Begin	End	Strand	Rep	Class	%	Total
HroSat02-1662	592	5.6	0.0	0.0	28	99	+	tRNA-Asp-GAY	tRNA	4,27	
HroSat02-1662	523	15.1	0.0	0.0	1344	1416	+	tRNA-Val-GTG	tRNA	4,33	
HroSat02-1662	244	26.4	7.1	1.1	1350	1441	+	SINE_FR2	SINE/tRNA-V-RTE	5,47	
HroSat02-1662	531	12.3	0.0	0.0	1523	1595	+	tRNA-Val-GTY	tRNA	4,33	18,41
HroSat03-372	359	20.6	0.0	0.0	89	151	C	tRNA-Val-GTG	tRNA	16,66	
HroSat03-372	458	16.7	0.0	0.0	290	361	C	tRNA-Asp-GAY	tRNA	19,08	35,75
HroSat05-515	411	16.1	0.0	0.0	32	93	+	tRNA-Val-GTY	tRNA	11,84	
HroSat05-515	225	15.4	4.9	0.0	66	104	C	Gypsy-11_XL-LTR	LTR/Gypsy	7,37	
HroSat05-515	375	21.4	0.0	0.0	178	247	+	tRNA-Val-GTG	tRNA	13,39	
HroSat05-515	371	18.8	0.0	0.0	335	398	+	tRNA-Asp-GAY	tRNA	12,23	44,85
HroSat06-1634	673	3.7	0.0	0.0	149	230	+	Nimb-4_DR	LINE/I	4,95	
HroSat06-1634	615	9.6	0.0	0.0	723	805	C	tRNA-Leu-CTG	tRNA	5,01	9,97
HroSat09-2961	1870	27.5	0.0	0.8	2	500	+	REX1-5_DR	LINE/Rex-Babar	16,81	
HroSat09-2961	337	27.1	0.0	0.0	678	747	+	tRNA-Ile-ATC	tRNA	2,33	
HroSat09-2961	591	9.6	0.0	0.0	868	940	C	tRNA-Ala-GCY	tRNA	2,43	
HroSat09-2961	603	8.1	0.0	0.0	1336	1409	+	tRNA-Ile-ATT	tRNA	2,46	
HroSat09-2961	563	18.1	0.0	1.1	1340	1434	+	TguSINE1	SINE/tRNA-CR1	3,17	
HroSat09-2961	3250	28.3	6.7	2.4	1449	2961	+	Rex1-3_EL	LINE/Rex-Babar	51,06	78,28
HroSat13-1440	629	0.0	0.0	0.0	7	79	C	tRNA-3_DRe	tRNA	5	
HroSat13-1440	323	26.6	1.3	11.5	614	787	+	SAT_LM	Satellite	12,01	17,01
HroSat17-2481	256	32.0	4.8	0.0	103	130	C	Ves2_ML	SINE/tRNA	1,08	
HroSat17-2481	623	4.2	0.0	0.0	131	202	C	tRNA-Lys-AAA	tRNA	2,86	
HroSat17-2481	398	16.7	1.3	4.9	140	221	C	tRNA-1_DRe	tRNA	3,26	
HroSat17-2481	611	5.6	0.0	0.0	1104	1175	+	tRNA-Trp-TGG	tRNA	2,86	
HroSat17-2481	275	26.1	0.0	0.0	1336	1404	C	tRNA-Lys-AAA	tRNA	2,74	
HroSat17-2481	333	25.7	0.0	1.3	1622	1696	C	tRNA-Gly-GGA	tRNA	2,98	
HroSat17-2481	628	4.2	0.0	0.0	1840	1911	C	tRNA-Gly-GGA	tRNA	2,86	18,66
HroSat18-1194	281	23.1	0.0	0.0	212	276	C	SINE2-1_SSc	SINE/tRNA	5,36	
HroSat18-1194	471	20.2	1.0	3.9	791	893	C	tRNA-3_DRe	tRNA	8,54	
HroSat18-1194	620	4.2	0.0	0.0	1109	1180	C	tRNA-3_DRe	tRNA	5,94	19,84
HroSat19-815	379	24.7	1.2	0.0	12	25	C	DNA-1_Gav	DNA	1,59	
HroSat19-815	564	8.4	0.0	0.0	26	96	C	tRNA-Gly-GGY	tRNA	8,58	10,18
HroSat23-576	596	8.8	0.0	0.0	60	139	+	tRNA-1_DRe	tRNA	13,71	
HroSat23-576	665	0.0	0.0	0.0	375	448	C	tRNA-Asn-AAC	tRNA	12,67	26,38

Supplementary Table 6

Query	SW	Percentage Div	Del	Ins	Position Begin	End	Strand	Rep	Class	%	Total
HduSat01-2707	355	24.7	9.3	2.0	9	157	C	Penelope-5_XT	LINE/Penelope	5,47	
HduSat01-2707	5420	30.2	2.3	2.6	305	2707	C	Penelope-5_XT	LINE/Penelope	88,73	94,20
HduSat03-2143	531	15.1	0.0	0.0	1532	1604	+	tRNA-Val-GTG	tRNA	3,36	
HduSat03-2143	268	25.3	7.1	1.1	1538	1629	+	SINE_FR2	SINE/tRNA-V-RTE	4,25	
HduSat03-2143	323	23.6	1.4	0.0	1711	1782	+	tRNA-Val-GTY	tRNA	3,31	
HduSat03-2143	404	18.3	0.0	0.0	2073	2143	+	tRNA-Asp-GAY	tRNA	3,27	14,19
HduSat08-1754	569	10.8	0.0	0.0	139	212	C	tRNA-Ile-ATT	tRNA	4,16	
HduSat08-1754	285	28.4	3.6	1.2	148	229	C	Mariner-N4_SSa	DNA/TcMar-ISRm11	4,62	
HduSat08-1754	264	21.9	4.7	2.4	596	606	C	tRNA-2_DRe	tRNA	0,57	
HduSat08-1754	591	9.6	0.0	0.0	607	679	+	tRNA-Ala-GCY	tRNA	4,10	
HduSat08-1754	364	25.7	0.0	0.0	800	869	C	tRNA-Ile-ATC	tRNA	3,93	
HduSat08-1754	1370	30.6	3.9	3.2	990	1732	C	Rex1-3_EL	LINE/Rex-Babar	42,30	59,69
HduSat10-1449	318	25.6	1.3	1.3	483	561	C	SAT_LM	Satellite	5,38	
HduSat10-1449	605	2.7	0.0	0.0	1197	1269	+	tRNA-3_DRe	tRNA	4,97	
HduSat10-1449	237	27.5	0.0	2.4	1198	1279	C	LTR10A_BT	LTR/ERV1	5,59	15,94
HduSat11-815	458	16.4	0.0	0.0	742	814	+	tRNA-Gly-GGY	tRNA	8,83	8,83
HduSat12-1670	661	4.9	0.0	0.0	355	436	C	Nimb-4_DR	LINE/I	4,85	
HduSat12-1670	340	19.2	13.1	0.0	563	635	+	tRNA-Ser-AGY	tRNA	4,31	
HduSat12-1670	615	9.6	0.0	0.0	1435	1517	+	tRNA-Leu-CTG	tRNA	4,91	14,07
HduSat17-150	279	21.2	3.9	2.0	43	143	C	Helitron-2_DR	RC/Helitron	66,67	66,67

Supplementary Table 7

Query	SW	Percentage Div	Del	Ins	Position Begin	End	Strand	Rep	Class	%	Total
HpuSat04-2707	355	21.8	7.3	2.3	4	183	C	Penelope-5_XT	LINE/Penelope	6,61	
HpuSat04-2707	5241	30.8	2.2	2.5	331	2707	C	Penelope-5_XT	LINE/Penelope	87,77	94,38
HpuSat07-2750	482	5.2	0.0	0.0	1	58	C	tRNA-Ile-ATT	tRNA	2,07	
HpuSat07-2750	541	13.7	0.0	0.0	462	534	+	tRNA-Ala-GCY	tRNA	2,62	
HpuSat07-2750	275	20.6	1.6	0.0	910	972	C	REX1-5_DR	LINE/Rex-Babar	2,25	
HpuSat07-2750	4491	28.4	3.8	1.8	984	2595	C	Rex1-3_EL	LINE/Rex-Babar	58,58	65,53
HpuSat08-1546	286	27.7	0.0	0.0	555	619	+	SAT_LM	Satellite	4,14	
HpuSat08-1546	656	3.5	0.0	2.3	781	867	C	tRNA-3_DRe	tRNA	5,56	9,70
HpuSat10-781	604	4.2	0.0	0.0	557	627	C	tRNA-Gly-GGY	tRNA	8,96	
HpuSat10-781	287	17.4	4.2	2.8	565	635	C	Bov-tA2	SINE/tRNA-Core-RTE	8,96	17,93
HpuSat12-1973	646	4.1	0.0	0.0	1475	1547	+	tRNA-Val-GTG	tRNA	3,65	
HpuSat12-1973	228	27.8	5.3	2.2	1481	1572	+	SINE_TE	SINE/tRNA-V	4,61	
HpuSat12-1973	569	9.6	0.0	0.0	1655	1727	+	tRNA-Val-GTG	tRNA	3,65	
HpuSat12-1973	592	5.6	0.0	0.0	1821	1892	+	tRNA-Asp-GAY	tRNA	3,60	15,51
HpuSat19-794	635	2.8	0.0	0.0	159	230	+	tRNA-Arg-AGG	tRNA	8,94	
HpuSat19-794	233	21.5	5.8	4.4	239	306	+	tRNA-Met-i	tRNA	8,44	17,38

Supplementary Table 8. Matched paired t-test. Different columns indicate the following data: Student t test (t Stat); degree of freedom (df); probability (p)

Pairs	t Stat	df	p
Hvi01_Hro02	6.548	40	4.013E-08
Hvi01_Hdu03	7.502	40	1.890E-09
Hvi01_Hpu12	7.664	40	1.134E-09
Hro02_Hdu03	5.435	40	1.466E-06
Hro02_Hpu12	6.505	40	4.620E-08
Hdu03_Hpu12	4.725	40	1.417E-05
Hvi03_Hpu05	-3.091	40	0.002
Hvi04_Hro09	1.186	40	0.121
Hvi04_Hdu08	6.207	40	1.210E-07
Hvi04_Hpu07	-4.891	40	8.380E-06
Hro09_Hdu08	4.912	40	7.825E-06
Hro09_Hpu07	-4.097	40	9.922E-05
Hdu08_Hpu07	-5.613	40	8.273E-07
Hvi05_Hro16	3.748	40	0.0003
Hvi05_Hdu06	3.433	40	0.0007
Hvi05_Hpu02	-2.874	40	0.003
Hro16_Hdu06	-3.286	40	0.001
Hro16_Hpu02	-2.917	40	0.003
Hdu06_Hpu02	-2.887	40	0.003

Pairs	t Stat	df	p
Hvi08_Hro14	-0.830	40	0.205
Hro04_Hvi13	2.322	40	0.012
Hro04_Hdu09	2.098	40	0.021
Hvi13_Hdu09	-2.081	40	0.022
Hvi14_Hro08	-3.275	40	0.001
Hvi14_Hdu02	-3.415	40	0.0007
Hro08_Hdu02	-2.627	40	0.006
Hro11_Hvi18	2.269	40	0.014
Hro11_Hdu15	2.388	40	0.011
Hvi18_Hdu15	-1.862	40	0.035
Hvi19_Hro18	-2.792	40	0.004

Supplementary Table 9. Consensus turnover rate (CTR) of satDNA sequences between *Harttia villasboas* (HviSatDNA) and *H. punctata* (HpuSatDNA).

HviSatDNA	HpuSatDNA	CTR
HviSat01-2531	0.189	0.54×10^{-8}
HviSat02-2707	0.025	0.07×10^{-8}
HviSat03-997	0.296	0.85×10^{-8}
HviSat04-2959	0.180	0.51×10^{-8}
HviSat05-177	0.163	0.47×10^{-8}
HviSat07-93	0.316	0.90×10^{-8}
HviSat09-815	0.271	0.78×10^{-8}
HviSat10-1666	0.498	1.42×10^{-8}
HviSat11-1338	0.771	2.20×10^{-8}
HviSat12-144	0.007	0.2×10^{-8}
HviSat15-32	0.000	0
	**Average**	0.71×10^{-8}

Supplementary Table 10. Summary of PCR and FISH results according to the distribution on autosomes (A) or sex chromosome (X_1). Probes using F1R1 primers were called HviZP4, while those using F2R2 primers Hvi-non-ZP4, and F3R3 HviSat08-4011. Colored cells indicate positive results.

		**FISH HviZP4**	**FISH Hvi-nonZP4**		**PCR**			
**Species**	**SatDNA**	**A**	**X_1**	**A**	**X_1**	**HviZP4**	**Hvi-non-ZP4**	**HviSat08-4011**
H. villasboas* and *H. rondoni	HviSat08-4011 and HroSat14-4165							
H. duriventris	Non-identified							
H. punctata	Non-identified							

Supplementary Table 11. Geographical coordinates, diploid number (2n), sex chromosome system, and the number of individuals (n) analyzed in this study. The number before the species name corresponds to the same presented in Figure 1. Specimens were deposited in the Museum of Zoology of the University of São Paulo (MZUSP) and the Ichthyological Collection of the National Institute for Amazonian Research (INPA-ICT). Species highlighted with asterisks correspond to those whose satellitomes were generated, while others were used only for FISH experiments.

Species	2n	Locality	Coordinates	n	Voucher
*1 - *Harttia villasboas*	56♀55♂ X_1X_2Y	Rio Curuá, Altamira (PA)	8°44'09''S 54°57'46''W	34♀38♂	MZUSP 126599
*4 - *Harttia duriventris*	56♀55♂ X_1X_2Y	Canaã dos Carajás (PA)	6°30'06.5''S 50°02'35.5''W	08♀07♂	MZUSP 126598
*2 - *Harttia rondoni*	54♀♂ XY	Rio Curuá, Altamira (PA)	8°38'53''S 55°01'41''W	15♀14♂	MZUSP 127606
*6 - *Harttia punctata*	58♀57♂ X_1X_2Y	Formosa (PA)	15°19'25''S 47°25'26''W	10♀12♂	MZUSP 111385
5 - *Harttia dissidens*	54♀♂	Igarapé Tambor, Rurópolis (PA)	4°5'37.8''S 55°0'30.2''W	07♀25♂	INPA-ICT 059577
9 - *Harttia guianensis*	58♀♂	Rio Curuá, Alenquer (PA)	1°29'02.2''S 54°50'31.2''W	06♀10♂	INPA-ICT 059584
3 - *Harttia* sp. 3	54♀♂	Rio do Peixe, Altamira (PA)	08°39'20.7''S 55°09'24.1''W	11♀15♂	MZUSP 127605
7 - *Harttia kronei*	58♀♂	Rio Açungui, Campo Largo (PR)	25º22'44''S 49º39'0.8''W	10♀, 5♂	MZUSP 109783
8 - *Harttia intermontana*	52♀/53♂ XY1Y2	Piranga river, Carandaí (MG)	20°59'34.0''S 43°43'30.0''W	20♀, 13♂	MZUSP 126520

Supplementary Data 1. Satellitome catalogues of four *Harttia* species. For *H. villasboas* and *H. rondoni*, the characteristics of both female and male satellitomes are displayed, while *H. duriventris* and *H. punctata* were based only in male samples. satDNA = satellite DNA; A+T(%) = percentage of adenine and thymine nucleotides; AM = abundance in males; AF = abundance in females; M/F = difference between male and female catalogues; Σ = sum AM+AF; DivM = divergence in males; DivF = divergence in females.
