# Supplementary material for: Independent evolution of satellite DNA sequences in homologous sex chromosomes of Neotropical armored catfish (Harttia)
Source: Commun Biol. 2025 Mar 30;8:524. doi: 10.1038/s42003-025-07891-6 (PMC11955569; doi:10.1038/s42003-025-07891-6)
Supplement: Supplementary file 2 — Description of Additional Supplementary File [file 42003_2025_7891_MOESM2_ESM.pdf]

Description Of Additional Supplementary File

File name: Supplementary Data 1
Description: Satellitome catalogues of four Harttia species. For H. villasboas and H. rondoni, the characteristics of both female and male satellitomes are displayed, while H. duriventris and H. punctata were based only in male samples. satDNA = satellite DNA; A+T(%)= percentage of adenine and thymine nucleotides; AM = abundance in males; AF = abundance in females; M/F = difference between male and female catalogues; Σ = sum AM+AF; DivM = divergence in males; DivF = divergence in females.
